# Cholesterol as a modulator of cannabinoid receptor CB_2_ signaling

**DOI:** 10.1038/s41598-021-83245-6

**Published:** 2021-02-12

**Authors:** Alexei Yeliseev, Malliga R. Iyer, Thomas T. Joseph, Nathan J. Coffey, Resat Cinar, Lioudmila Zoubak, George Kunos, Klaus Gawrisch

**Affiliations:** 1 National Institute on Alcohol Abuse and Alcoholism, National Institutes of Health, Bethesda, MD 20852 USA; 2 Department of Anesthesiology and Critical Care, Perelman School of Medicine, University of Pennsylvania, Philadelphia, PA 19104 USA

**Keywords:** GTP-binding protein regulators, Membrane proteins, Lipids, Proteins, Structural biology, Biochemistry, Biological techniques, Biophysics, Drug discovery, Medical research

## Abstract

Signaling through integral membrane G protein-coupled receptors (GPCRs) is influenced by lipid composition of cell membranes. By using novel high affinity ligands of human cannabinoid receptor CB_2_, we demonstrate that cholesterol increases basal activation levels of the receptor and alters the pharmacological categorization of these ligands. Our results revealed that (2-(6-chloro-2-((2,2,3,3-tetramethylcyclopropane-1-carbonyl)imino)benzo[*d*]thiazol-3(2*H*)-yl)ethyl acetate ligand (MRI-2646) acts as a partial agonist of CB_2_ in membranes devoid of cholesterol and as a neutral antagonist or a partial inverse agonist in cholesterol-containing membranes. The differential effects of a specific ligand on activation of CB_2_ in different types of membranes may have implications for screening of drug candidates in a search of modulators of GPCR activity. MD simulation suggests that cholesterol exerts an allosteric effect on the intracellular regions of the receptor that interact with the G-protein complex thereby altering the recruitment of G protein.

## Introduction

Lipid composition of membranes plays an important role in modulation of G protein-coupled receptors (GPCR)^1–4^. In particular, cholesterol content varies significantly among different types of cell membranes, and was shown to influence the ligand-induced signal transduction through GPCRs^5–8^. Early stage pharmacological characterization of GPCR ligands typically involves screening of prospective drug candidates in cell- and membrane-based assays^9^. Hence, it is important to understand how a physiological response of the target receptor to small molecule ligands is modulated by the lipid composition of membranes.

Membranes of mammalian cells typically used for expression of recombinant GPCR for cell- and membrane-based assays may contain significant amount of cholesterol^10^. Plasma membranes of human embryonic kidney (HEK293) and Chinese hamster ovary (CHO) cells were reported to contain as much as 25–40 mol% of cholesterol relative to phospholipid^10–12^. The lipid and cholesterol content of cultured mammalian cells fluctuates depending on nutrient composition of growth media, temperature of cultivation, age of cell culture and other growth parameters^10^. Other commonly used expression hosts such as *Escherichia coli*^13,14^, *Spodoptera frugiperda* (Sf9), and *Trichoplusia ni* (Tn) synthesize only trace amounts of cholesterol or do not produce it at all^11^.

A large proportion of pharmacological drugs, currently on the market or under development, target membrane proteins. Since pharmacological profiles of drug candidates could be influenced by the properties of cell membranes harboring these receptors, variations in composition of the lipid bilayer may result in inconsistencies of the receptor response^15,16^. This is especially true for hydrophobic ligands targeting GPCR since interaction between the drug and the lipid may influence the pharmacological characteristics of the compound such as its binding affinity and selectivity^17–19^. While it was demonstrated that lipids and cholesterol can shift the response of the receptor to ligand binding, it is not well known if the pharmacological categorization of ligands, i.e. agonism, antagonism or inverse agonism, depends on composition of the lipid matrix. Here, we sought to elucidate whether the functional response of the cannabinoid receptor CB_2_ to a series of novel specific ligands is affected by the cholesterol content of membranes.

Cholesterol is critical for the formation of lateral domains (clusters)^20^, and it may induce negative curvature elastic stress in lipid bilayers^21^. The cholesterol-dependent increase in membrane stiffness increases the decay length of protein-induced perturbations in the lipid matrix^22^. Furthermore, cholesterol may directly interact with GPCR at sites identified in several receptors^23–26^. Cholesterol was reported to negatively modulate the activity of type 1 cannabinoid receptor (CB_1_) in nerve cells^27^. Furthermore, it was proposed that the CB_1_ receptor possesses a specific cholesterol binding site^28^. However, the activity of the structurally close cannabinoid receptor CB_2_ was reported to be not influenced by changes in membrane cholesterol content^29^. The CB_2_ receptor is known for its role as a regulator of inflammation and is commonly found in tissues containing variable levels of cholesterol^30^. Therefore, it is important to understand if CB_2_ is sensitive to variation in cholesterol content.

Here, we examined the effects of cholesterol on CB_2_ by analyzing G protein activation by this receptor in response to novel, rationally designed CB_2_ ligands (MRI-2646, MRI-2654, MRI-2687, MRI-2653, MRI-2659). We will demonstrate that cholesterol increases the basal activation levels of CB_2_ receptor, thereby altering pharmacological classification of these ligands from inverse agonists to partial agonists. We also use MD simulation to probe structural effects of cholesterol and ligands.

## Results

Based on the rational modification of the A-836339 thiazole scaffold, some of us previously reported that the synthetic compounds MRI-2687 and MRI-2594 show contrasting activity on the CB_2_ receptor^31^. The two compounds only differ in arm 1 featuring the extended central 6-methylbenzothiazole ring in MRI-2687 and 4,5-dimethylthiazole ring in MRI-2594, respectively. Unexpectedly, MRI-2687 behaved as an inverse agonist, whereas MRI-2594 acted as an agonist on CB_2_ (similar to A-836339)^31,32^. Detailed molecular docking analysis showed that these two ligands adopt similar binding poses within CB_2_ wherein the different central rings reside in the same position as arm 1 of the receptor bound AM10257 CB_2_ crystal structure^31^. The arm 1 of MRI-2687 along with its 6-Me substitution forms π–π interactions with the side chain of Trp258^6.48^ and confines its conformation to a similar rotamer as in the CB_2_-AM10257 structure. As opposed to MRI-2687, the lack of a large substituent on arm 1 of MRI-2594 prevents it from extending sufficiently deep to constrain the conformation of Trp258^6.48^ and allows its unrestrained movement and, thereby, leading to receptor activation. The proposed differential interactions with the toggle switch residue Trp258^6.48^ prompted us to investigate the effect of these novel ligands on CB_2_ receptor further in various cell-and membrane-based assays.

**Figure 1:**
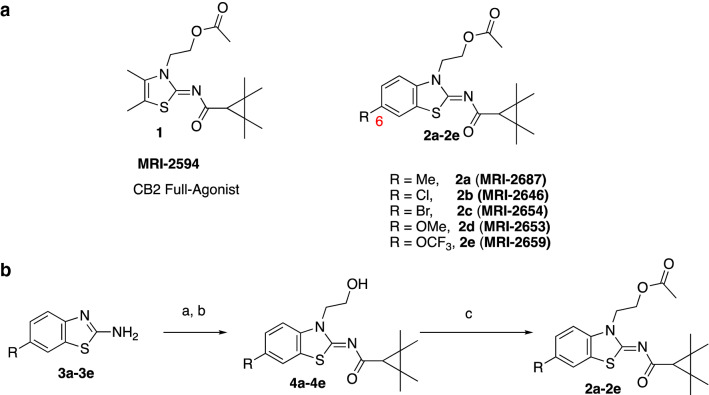
Benzothiazole-based CB_2_ ligands. (**A**) Structure of benzothiazole-based CB_2_ ligands. (**B**) Synthesis scheme for ligands, Reagents and conditions: a. 2-bromoethanol 90 °C; b. BOP, 2,2,3,3-tetramethylcyclopropane carboxylic acid. c. Acetyl Chloride.

To assess the function of CB_2_ in vitro, we measured the activation of the receptor by quantifying the rates of nucleotide exchange on the G_α_ subunit of G protein that interacts with CB_2_. The full agonist CP-55,940 is not well suited for studying modulatory effects of the lipid bilayer on receptor function since its strong activating effect may mask moderate effects exerted by the lipid matrix. This is an important consideration since, unlike full synthetic agonists, many endogenous agonists of GPCR only partially activate these receptors. We hypothesized that our novel series of high-affinity ligands would allow detection of moderate modulating effects of the lipid matrix on CB_2_ activation. Specifically, we sought to investigate the role of different substitutions at the 6-position of the benzothiazole ring in this series of ligands. Elaborating on our earlier approach^31^, we synthesized structurally-related compounds (Fig. 1) and evaluated their effects on CB_2_ receptor activity.

In this study, we used the recently developed cannabinoid ligands which were synthesized as shown in Fig. 1A. The R group at the 6-position of the benzothiazole were varied to include Me, OMe, OCF_3_, Cl, and Br, synthesized as shown in Fig. 1B.

**Figure 2:**
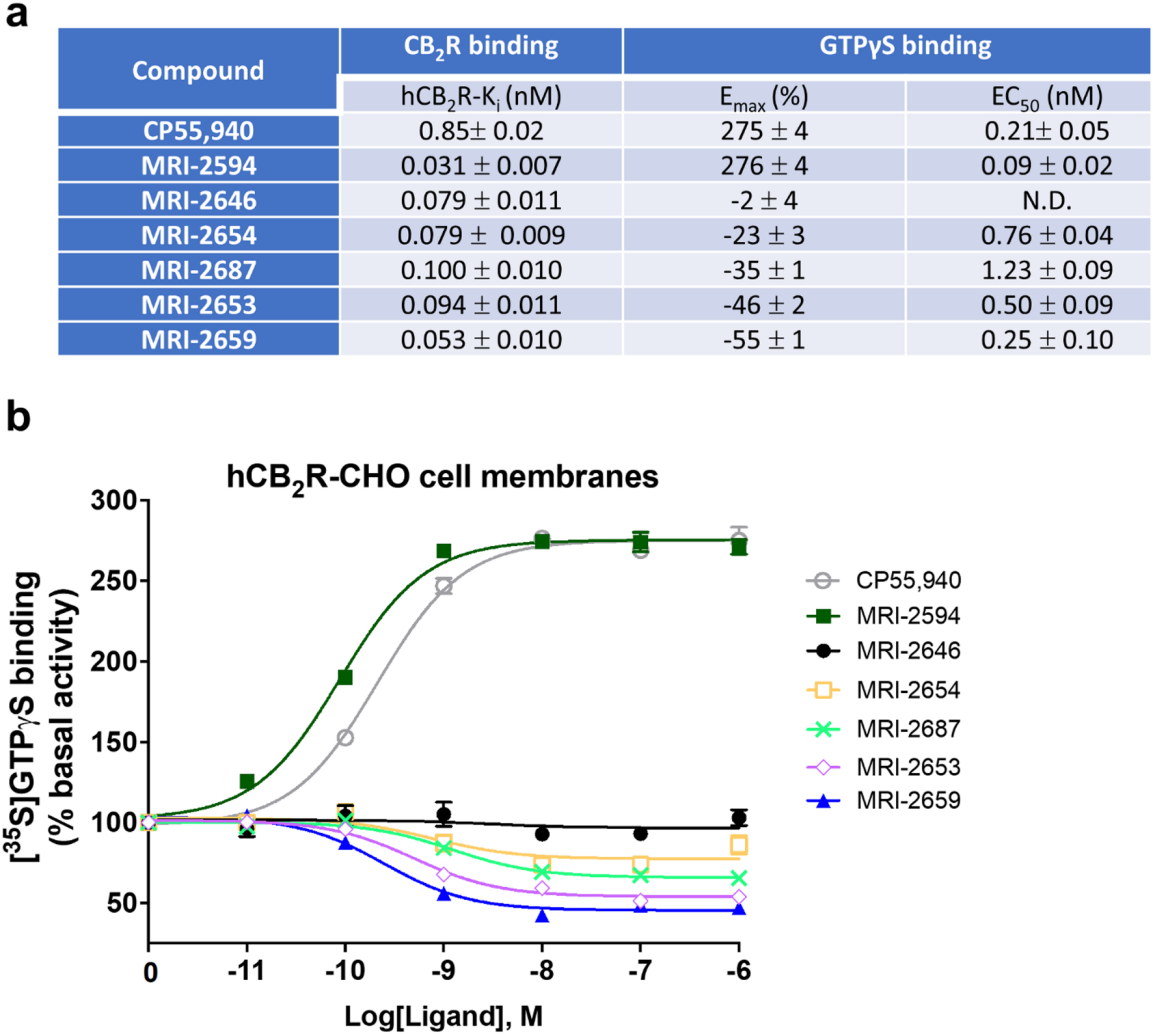
Affinity and functional effects of novel MRI ligands on CB_2_ receptor in CHO cell membranes. (**a**) binding affinities (nM) and E_max_ (% and nM) of [^35^S]-GTPγS binding to CHO membranes (PerkinElmer, Cat. No ES111-M400UA) expressing hCB_2_. (**b**) [^35^S]-GTPγS binding to membranes as a factor of ligand concentration. Binding of [^35^S]-GTPγS was determined as described in “Methods”. Non-specific binding was defined as 0% activity. The assay of GTPγS non-specific binding contains non-radioactive GTPγS.

The affinity of the MRI ligands for CB_2_ receptor was determined to be in 0.053–0.1 nM range in hCB_2_-expressing-CHO cell membranes by a displacement binding experiment (Fig. 2a).

Then, we assessed the functional effect of the compounds on G-protein signaling in [^35^S]-GTPγS binding assays using hCB_2_-expressing-CHO cell membranes obtained from PerkinElmer (see “Methods”). MRI-2646 had no effect on basal signaling (Fig. 2b) whereas MRI-2654, MRI-2687, MRI-2653, and MRI-2659 reduced basal signaling to 55% with high potency (Fig. 2a) as a function of inverse agonism. Unlike other structural analogues, MRI-2594 demonstrated full agonism with high affinity (hCB_2_R, Ki = 0.031 nM) and potency (EC_50_: 0.09 nM) (Fig. 2a). We were intrigued by the seemingly neutral antagonist activity of MRI-2646 in CHO cells even though its structural features were in line with the other benzothiazole ligands which behaved as inverse agonist. Hence, in the present work, we used this novel, high affinity cannabinoid ligand to explore the role of lipid environment in determining functional activity.

Based on our preliminary studies, we concluded that ligands such as MRI-2646 may act either as a weak partial agonist or a neutral antagonist, depending on the cell type used in the assay. To better understand the reason for these discrepancies, we assessed the activation of CB_2_ receptor by the in vitro G protein activation assay that measures the rates of nucleotide exchange on the G_α_ subunit of G protein (GEF assay) as described previously^33,34^. The assay reports the rates of formation of the complex of [^35^S]-GTPγS, a homolog of GTP, with the G_α_ subunit of G protein. Typical assay conditions require small (nanogram) quantities of the receptor protein either in cell membranes or reconstituted into lipid bilayers. The readout of the assay is the amount of the non-hydrolysable complex of G_α_ with [^35^S]-GTPγS which, under selected experimental conditions, is proportional to the amount of activated receptor in the assay.

For convenience, the results of the GEF assay are normalized such that activation of CB_2_ in the presence of CP-55,940 was set to 100%, and residual activity in the presence of saturating concentrations of the full inverse agonist SR-144,528 was set to 0%.

**Figure 3:**
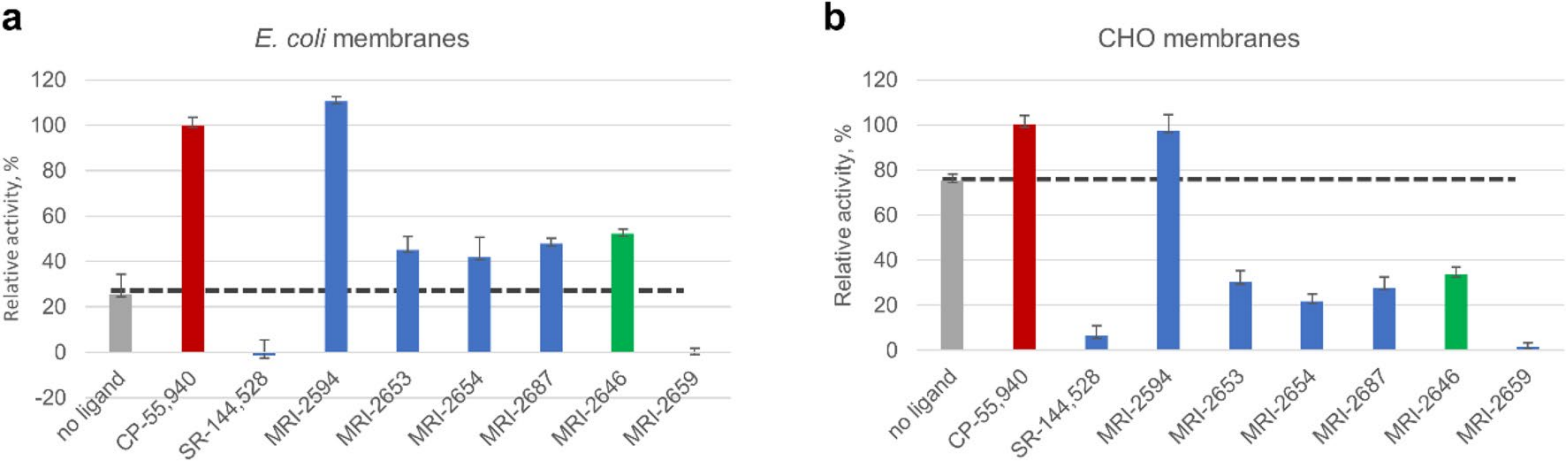
GEF of CB_2_ expressed in: (**a**) *E. coli* membranes and (**b**) CHO membranes (Millipore EMD). 2 μg of total protein per assay. Each point represents an average of four independent measurements (n = 4). Ligands were added at a concentration of 2 μM to ensure saturation of the receptor, and G protein was added as described in “Methods”. The dotted line indicates the rates of activation of G protein in the absence of a ligand. The rates of activation with CP-55,9040 are set to 100%, and rates of activation in the presence of SR-144,528 to 0%.

We first compared two different types of membranes: *E. coli* BL21 (DE3) and a commercially available preparation of CHO cell membranes expressing CB_2_ (Millipore EMD, Cat. No HTS020M) in the GEF assay (Fig. 3). Depending on the source of membranes, a significant difference in activation behavior of CB_2_ was observed. In this assay, the tested ligands (with the exception of the previously described full agonist MRI-2594^31^ and strong inverse agonist MRI-2659) behaved as partial agonists of the CB_2_ receptor in *E. coli* membranes. However, these same ligands acted as partial inverse agonists on CB_2_ expressed in CHO cells (Fig. 3). The activities of the ligands relative to each other, which were roughly inversely correlated to the ligand substituent size, were not greatly changed, and correlated roughly to the R-group size.

**Figure 4:**
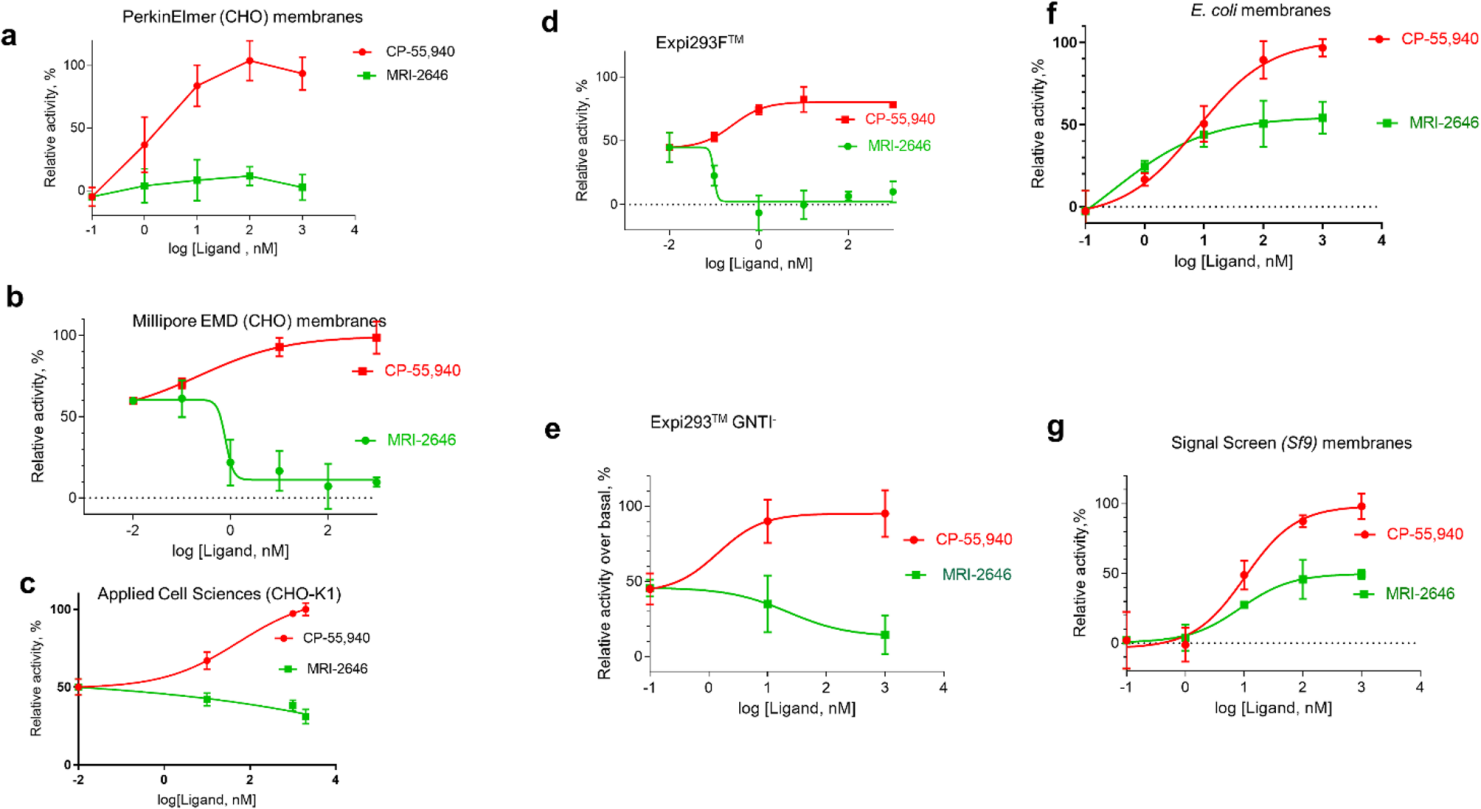
GEF on CB_2_ expressed in different cell lines. An amount of 2 μg of membrane protein per assay was used. Each data points represents an average of four independent measurements (n = 4) with error rates indicated by the bars. 100% represents full activation in the presence of 2 μM of CP-55,940, and 0%–with 2 μM SR-144,528.

Intrigued by these observations, we compared several other available sources of membranes expressing CB_2_. MRI-2646 ligand was selected as a representative of a cohort of related benzothiazole ligands for all subsequent measurements since it activated CB_2_ in *E. coli* cell membranes the most while being a partial inverse agonist of CB_2_ in CHO membranes. Several preparations of membranes expressing CB_2_ were compared (Fig. 4). While MRI-2646 behaved as a neutral antagonist of CB_2_ in membranes from CHO cells obtained from PerkinElmer, it acted as a partial inverse agonist in two other commercial preparations of CB_2_ in CHO membranes procured from EMD Millipore and Applied Cell Sciences (CHO-K1 membranes). Likewise, MRI-2646 was a partial inverse agonist of CB_2_ expressed in membranes Expi293F and Expi293F GNTI^-^ cells. On the other hand, this ligand was a partial agonist of CB_2_ expressed in baculovirus infected insect Sf9 cells and in *E. coli* BL21 (DE3) cells.

Therefore, MRI-2646 exerts differential effects on CB_2_: in mammalian cell membranes this ligand acts as an inverse agonist or neutral antagonist while in bacterial- and insect-cell membranes it behaves as a partial agonist of CB_2_ receptor. Likely contributing factors to such discrepancies may include: (i) differences in lipid composition (in particular, cholesterol content) between mammalian, insect, and bacterial membranes; (ii) different pattern of post-translational modifications (palmitoylation, glycosylation) of receptor molecules expressed in different expression hosts; (iii) differences in expression levels of the receptor and densities of ligand-binding sites in membrane preparations from different sources, and (iv) composition of endogenous G proteins in membrane preparations. The endogenous membrane-associated G protein contained in preparations of CHO, HEK and Sf9 cell membranes expressing CB_2_ contributes to the GEF signal. *E. coli* cells do not produce G protein. The subtle role of the sterics and electronics of the 6-Cl substituent in this intricate modulatory mechanism cannot be ruled out either.

### Effects of lipid composition

We considered the effects of lipid composition of CB_2_-containing membranes obtained from different expression hosts. Specifically, the CHO cell membranes are known to contain high concentrations of cholesterol, unlike the *E. coli* cell membranes that are devoid of cholesterol^12^. We quantified the relative content of lipids and cholesterol in several preparations of membranes of mammalian, insect, and *E. coli* cells (Supporting Fig. 1). Lipids were extracted from membranes, and their composition determined by ^1^H-NMR as described in Legend to Supporting Fig. 1. Consistent with the previously published data, cholesterol was not detected in membrane preparations from *E. coli* and from Sf9 cells^11,35–37^ while membrane preps obtained from CHO cells and suspension culture of HEK Expi293F expressing CB_2_ contained 39% and 26% cholesterol relative to phospholipids, respectively^10,38^. While the bacterial-, insect- and mammalian cell membranes differ significantly not only in content of cholesterol but also in composition of phospholipids^10,11,13^, a variability in cholesterol content between membranes from different expression cell lines correlates strongly with the signaling pattern of CB_2_ activated by the novel ligand. Therefore, we hypothesized that the cholesterol content of membranes affects the activation of the cannabinoid receptor CB_2_.

### Endogenous vs. exogenous G protein

Besides lipids, the content of endogenous G proteins in cell membrane preparations from different sources may also affect the readout of the [^35^S]-GTPγS binding and the GEF G-protein activation assays used in this study (see “Methods”). The [^35^S]-GTPγS binding assay measures the binding of the radiolabeled nucleotide analogue to the endogenous G protein that is already present in membrane preparations while the GEF assay relies on the exogenous G protein subunits of G_αi1_ and G_β1γ2_ added in large excess relative to receptor. Therefore, the GEF assay typically affords a good signal-to-noise ratio and enables comparison of multiple samples at standardized conditions. The addition of G protein is necessary to analyze the activation of CB_2_ in *E. coli* membranes since these membranes do not contain endogenous G protein. On the other hand, the membranes obtained from mammalian and insect cell cultures contain endogenous G proteins and, therefore in these membranes, the GEF assay reports on the rates of activation of a combined pool of endogenous as well as exogenous G protein.

**Figure 5:**
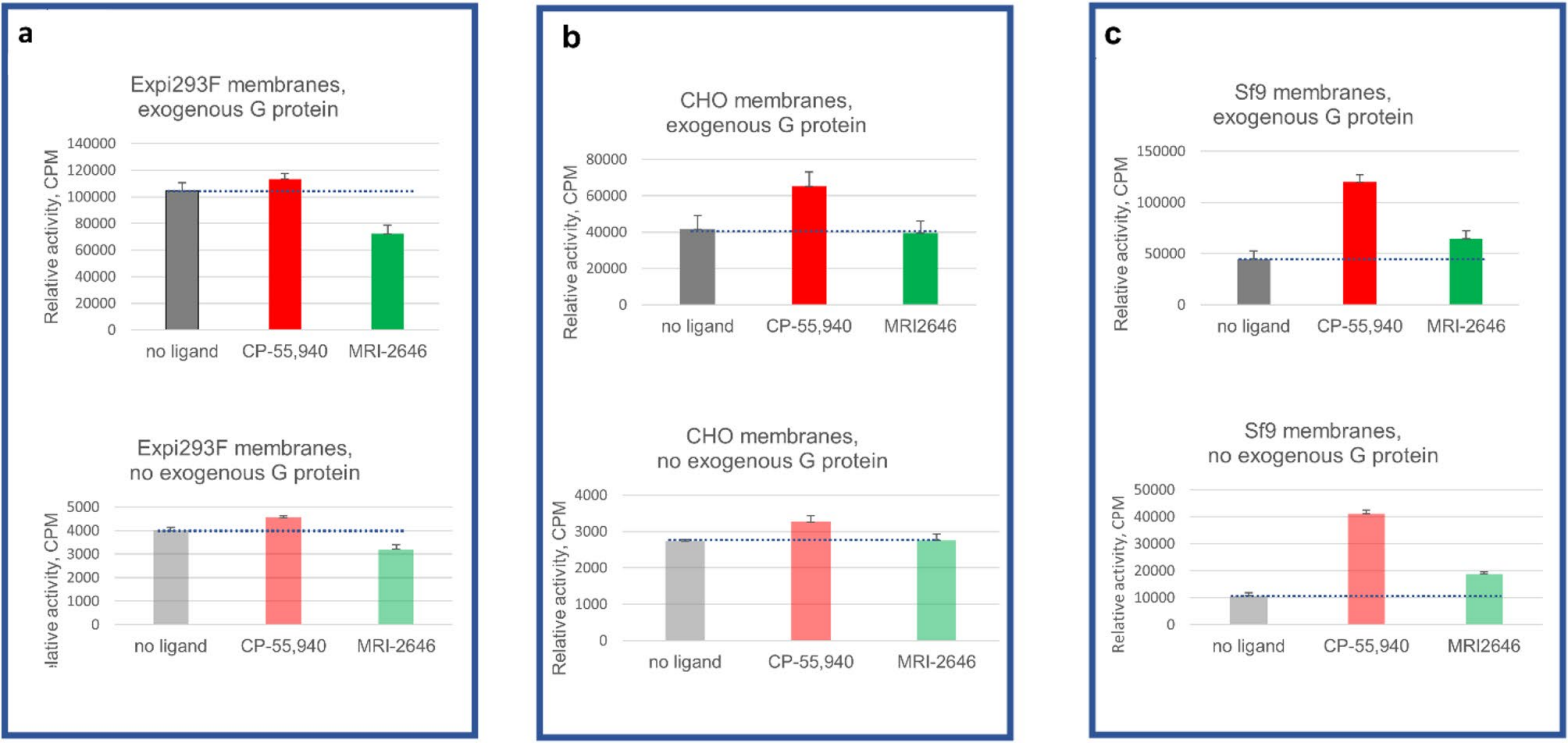
GEF analysis of three membrane preparations with or without supplementation with exogenous G protein. (**a**) Expi293F membranes; (**b**) CHO membranes; (**c**) Sf9 membranes. Bars represent an average of four independent measurements (n = 4). Dotted lines represent the rates of activation of G protein in the absence of a ligand.

To assess the contribution of endogenous G protein to the total GEF signal, we performed the GEF assay on membranes of Expi293F, CHO (Perkin Elmer) and Sf9, in the absence as well as in the presence of exogenous G protein (Fig. 5).

As expected, in the absence of exogenous G protein the magnitude of the signal was significantly lower (three to tenfold) compared to the standard GEF assay. Yet, the pattern of activation of CB_2_ by the MRI-2646 was similar in assays performed with- and without addition of exogenous G protein. While in membrane preps from Expi293F cells, MRI-2646 acted as an inverse agonist of CB_2_ (Fig. 5a); in CHO cell membranes (PerkinElmer) it behaved as a neutral antagonist (Fig. 5b); and in membranes of Sf9 cells expressing CB_2,_ it acted as a partial agonist (Fig. 5c).

### Receptor density in membranes

We next examined the density of ligand binding sites (by saturation ^3^H-CP-55,940 radioligand binding assay, Supporting Fig. 2). The density of the ligand binding sites varied between ~ 22 and 50 pmol/ mg of membrane protein. However, there was no correlation between the density of the ligand binding sites and behavior of the MRI-2646 ligand as an inverse agonist, neutral antagonist or partial agonist of CB_2_. Thus, the differential pattern of activation of CB_2_ by the MRI-2646 cannot be explained by differences in expression levels and density of binding sites of receptor in membrane preparations.

The results above suggest that the functional effect of MRI-2646 on CB_2_ varies following the same pattern as the content of cholesterol in membranes of cell lines expressing CB_2_. While in membranes devoid of cholesterol, the MRI-2646 is a partial agonist, and, in cholesterol-containing membranes it acts either as an inverse agonist or a neutral antagonist of CB_2_.

### Treatment with methyl-β-cyclodextrin

The content of cholesterol in cell membrane preparations can be altered by pre-treatment with methyl-β-cyclodextrin (MβCD)^8,39^ (Supporting Fig. 3). In membranes of Expi293F cells expressing CB_2_, treated with 20 mM MβCD, the rates of activation of G protein on CB_2_ decreased by about twofold although the levels of the CB_2_ receptor in these membranes were unchanged (Supporting Fig. 3b). In the MβCD-treated membranes, the activity of CB_2_ in the presence of MRI-2646 was higher than the basal signaling in the absence of ligands, indicating that MRI-2646 ligand acted as a partial agonist of CB_2_ receptor at these conditions.

**Figure 6:**
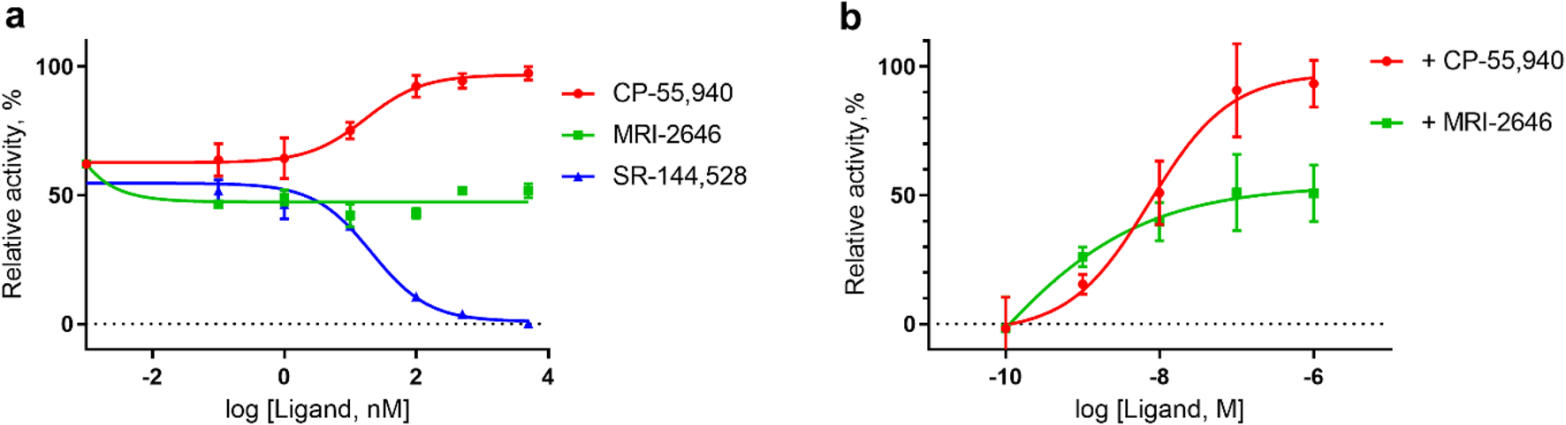
Effect of treatment with MβCD/Chol of *E. coli* membranes on activation of CB_2_ by synthetic ligands. (**a**) Membranes expressing CB_2_ were treated with 20 mM MβCD/Chol for 1 h at 4 °C, washed with PBS, and the activation of CB_2_ in the presence of ligands determined by GEF; (**b**) control, untreated *E. coli* membranes expressing CB_2_. Each data point represents an average of four independent measurements (n = 4) with standard deviation indicated by vertical bars.

MβCD can also be used as a carrier of cholesterol in order to enrich cell membranes with cholesterol^40^. To test the effect of the exogenously added cholesterol on activation of CB_2_, we pre-treated the *E. coli* membranes expressing CB_2_ with a solution of MβCD/cholesterol, and measured the rates of activation of G protein (Fig. 6a). In the MβCD/cholesterol-treated membranes of *E. coli*, the MRI-2646 reproducibly acted as a neutral antagonist of CB_2_, while in the untreated membranes it exhibited agonistic effects (Fig. 6b). There was no noticeable change in the levels of CB_2_ in membranes, and the density of ligand binding sites did not change upon treatment with MβCD/cholesterol (Supporting Fig. 4a,b) These results provide a more direct proof that the activation of CB_2_ bound to MRI-2646 is modulated by cholesterol content of membranes.

### ***Liposome-reconstituted CB***_***2***_*** and post-translational modifications***

To assess the possible role of post-translational modifications of CB_2_ on its activation by the MRI ligands, the recombinant CB_2_ receptor was isolated from two different expression cell lines, *E. coli* BL21 (DE3)^41^ and Expi293F GNTI^-^^42^. The protein was purified and reconstituted into lipid bilayers containing 1-palmitoyl-2-oleoyl-glycero-3-phosphocholine (POPC) and, 1-palmitoyl-2-oleoyl-sn-glycero-3-phospho-(1′-rac-glycerol) (POPG) at a molar ratio of 3/1, either supplemented or not supplemented with cholesterol, as described in “Methods”^10,13^. We reported previously that the presence of phospholipids with a negatively charged headgroup stabilizes CB_2_ protein in lipid bilayers^43^. Therefore, purified CB_2_ was reconstituted into POPC/POPG (3/1, mol/mol) liposomes containing 0, 20 and 40 mol% cholesterol (reported as total content of lipids) as described in “Methods”. In one case, the protein purified from *E. coli* cells was reconstituted into lipids extracted from brain tissue (Avanti Polar Lipids). The ratio of protein-to-lipid in the resulting samples was in the range of 1:850 to1:1100 (mol/mol). The levels of protein, and the density of ligand binding sites for receptor preparations reconstituted in liposomes with different content of cholesterol varied only slightly (Supporting Fig. 5a,b).

**Figure 7:**
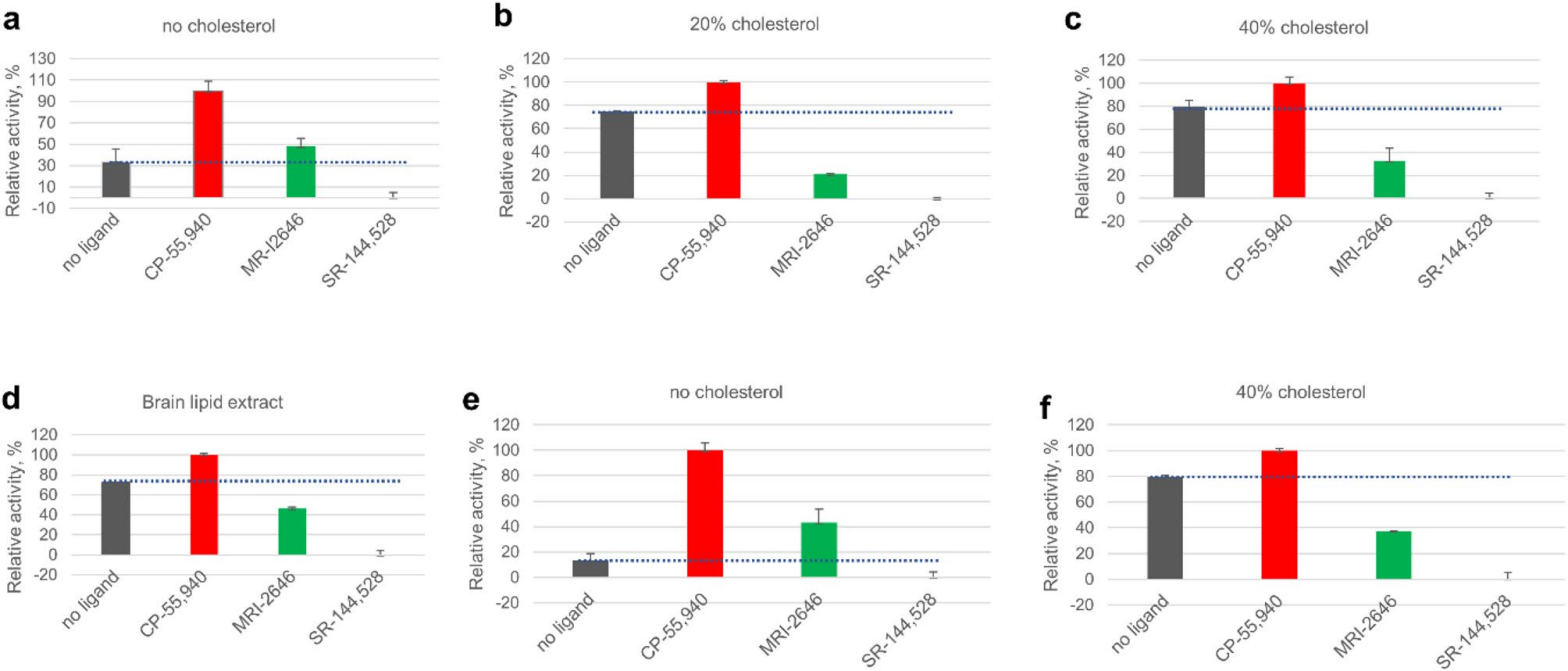
Activation of G protein on CB_2_ receptor reconstituted in liposomes. Liposome composition: 75% POPC, 25% POPG and cholesterol content relative to total phospholipids as indicated, or total lipids extracted from bovine brain, as indicated. Proteins were reconstituted at a protein-to-lipid ratio in the range 1:850 to 1:1100 mol/mol. (**a**–**d**) CB_2_ purified from *E. coli* cells. (**e**,**f**) CB_2_ purified from Expi293F GNTI^-^ cells. Average values of four independent measurements are plotted (n = 4). Dotted lines represent the rates of activation of G protein in the absence of a ligand.

The results of the GEF assay performed on these liposome-reconstituted CB_2_ samples demonstrate that the MRI-2646 ligand acts as a partial agonist of bacterially expressed CB_2_ in liposomes devoid of cholesterol (Fig. 7a). However, in the lipid matrix containing 20% or 40% of cholesterol or in liposomes composed of lipids extracted from brain tissues (Fig. 7b–d), the basal (without ligand) activation of CB_2_ receptor was increased. In all cholesterol-containing liposomes, including those containing the brain lipid extract, the MRI-2646 ligand acted as a partial inverse agonist of the receptor.

For comparison, the activation behavior of CB_2_ isolated from the Expi293F GNTI^-^ cells^42^ and reconstituted into liposomes was studied (Fig. 7e). In liposomes without cholesterol, the MRI-2646 acted as a partial agonist of CB_2_, similar to its action on the bacterially expressed protein. At the same time, MRI-2646 acted as a partial inverse agonist on HEK cell-expressed CB_2_ protein reconstituted into liposomes with 40% of cholesterol (Fig. 7f and Supporting Fig. 5, 6). These results corroborate the above described inverse agonism of MRI-2646 on CB_2_ in membrane preps containing cholesterol. Therefore, it can be concluded that cholesterol is involved in modulating the activation behavior of CB_2_. Post-translational modifications of CB_2_ do not seem to play a significant role in modulation the activation of the receptor by MRI-2646 in the presence of cholesterol.

### ***Structural effects of cholesterol on CB***_***2***_*** in molecular dynamics simulation***

Using molecular dynamics (MD) simulations, we evaluated the effect of membrane cholesterol on three of the ligands’ interaction with the known toggle switch residue Trp258^6.48^as well as the displacement and fluctuation of the ICL3-TM6 region of CB_2_, which would interact with the G-protein upon its recruitment. We hypothesized there would be a significant effect of membrane cholesterol on these regions in free CB_2_.We anticipated three alternatives for the effect of ligand type on the simulation results: (1) no obvious effect of ligand; (2) effects specific to the experimentally observed ligand pharmacological category; or (3) effects correlating mainly to ligand size. MRI-2646. MRI-2659, and MRI-2594 were simulated, chosen for their diversity.

**Figure 8:**
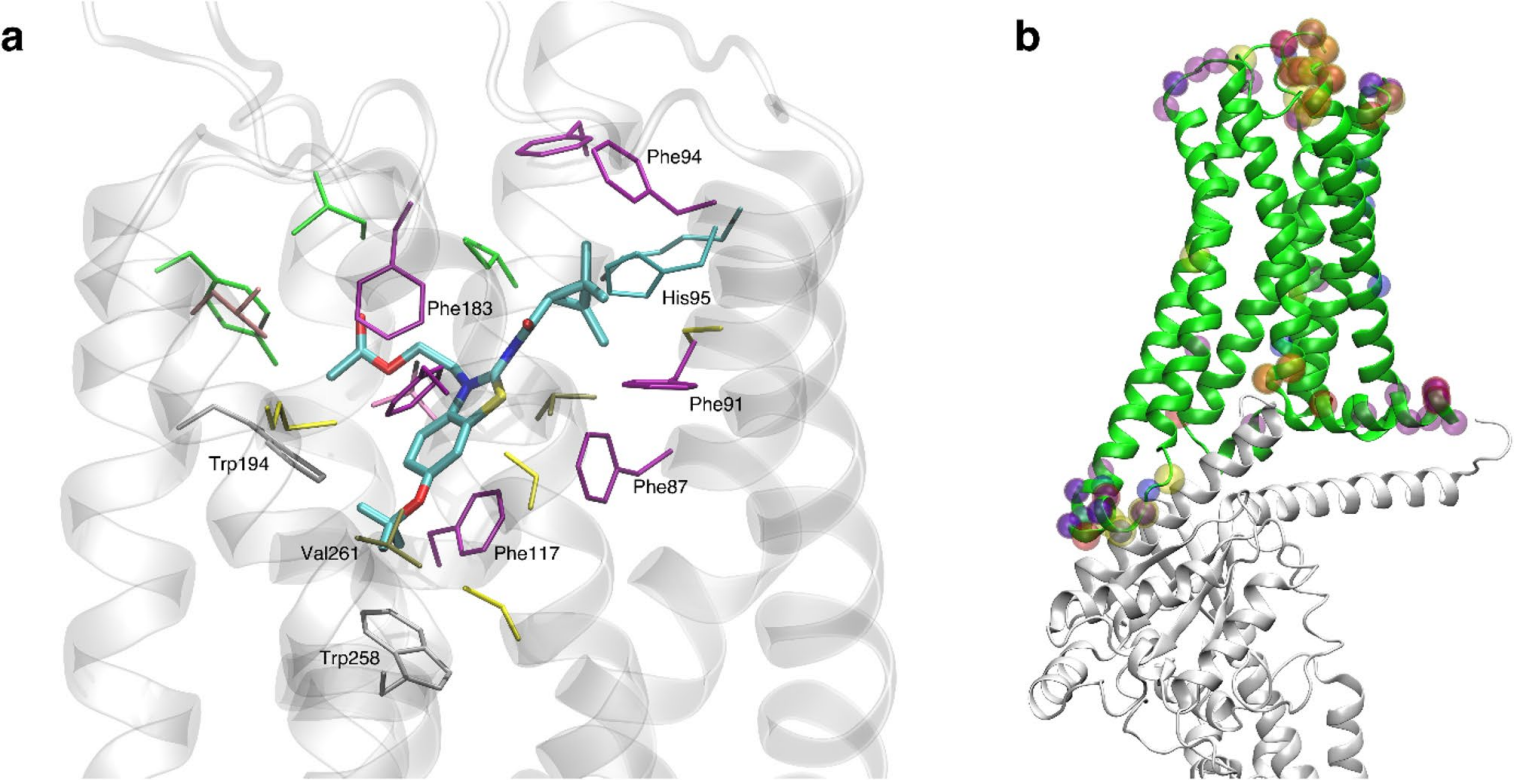
Depictions of ligand binding site and preferentially affected residues. (**a**) Representative snapshot of MRI-2659 in orthosteric binding site. MRI-2646 and MRI-2659 share common backbone position; (**b**) Cryo-EM structure (PDB: 6PT0)^44^. Those residues whose simulation average RMSD from the cryo-EM structure changed by > 1 Å as a function of 0% vs 40 mol% cholesterol are highlighted by spheres. Such residues from free and ligand-bound conditions (MRI-2594, MRI-2646, MRI-2659) are all shown. Note proximity to G_α_ subunit (grey). A number of these residues reside in the extracellular loops; the functional relevance is not known.

We conducted equilibrium all-atom molecular MD simulations of the cryo-EM structure of CB_2_ (PDB: 6PT0)^44^ with and without 40% membrane cholesterol and a POPC/POPG, 3/1, mol/mol ratio. The cholesterol molecules in the extracellular part of the TM5-6 region reported in the cryo-EM structure were retained, particularly since this is a known cholesterol binding site^45,46^. In both of these conditions, simulations were done with no bound ligand as well as with each of the three chosen ligands. Each ligand was placed respectively in the orthosteric site (Fig. 8) by analogy with the configuration of the structurally similar AM10257 ligand agonist present in the X-ray structure of CB_2_^31^. There were eight total simulations.

The R-groups of the ligands were in close proximity to the toggle switch residue Trp258 ^6.48^. In each simulation, the protein underwent an initial relaxation phase from the initial antagonist-bound cryo-EM conformation within 10 ns, as shown by evolution of whole protein RMSD over time (Supporting Fig. 7). Each ligand remained stable in its initial position throughout (Supporting Fig. 7). We discarded the first 50 ns of each production simulation, to include the relaxation phase, for an aggregated grand total of 2 µs of simulation trajectory analyzed across all conditions. Key binding site interactions are shown in Fig. 8a.

In GEF experiments, membrane cholesterol increased the constitutive activity of CB2, and the relative order of MRI-2594, MRI-2646, and MRI-2659 by activity was preserved, even though these ligands would be categorized differently relative to the benchmark of constitutive activity. Since G-protein recruitment is thought to depend on the toggle switch Trp258^6.48^, we hypothesized that the rotameric state of this residue would be differentially changed by ligand type, particularly since the ligand substituent is in close proximity to it.

**Figure 9:**
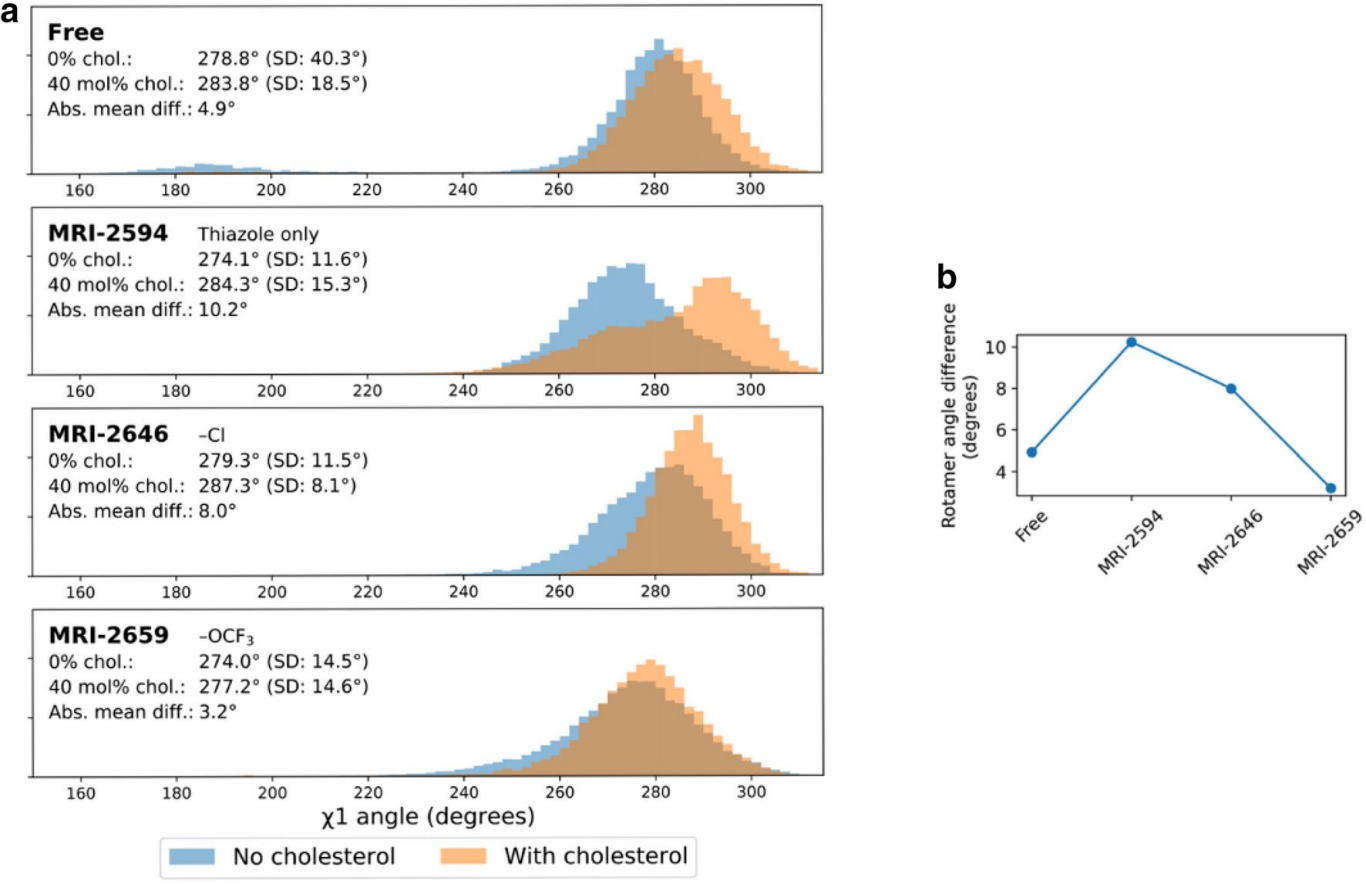
Difference in Trp258^6.48^ rotamer angle related to presence of cholesterol. (**a**) Trp258^6.48^ χ^1^ rotamer angle distributions with 0% and 40 mol% cholesterol. (**b**) Difference in mean Trp258^6.48^ χ^1^ rotamer angle between no cholesterol vs 40 mol% cholesterol conditions; green (negative change) dashed line. TM domains are annotated, with up and down arrows indicating direction of helix, with up from intracellular to extracellular.

We found that the distribution of Trp258^6.48^ side-chain χ^1^ rotamer angles (from the N–Cα–Cβ–Cγ dihedral) during the simulations varied by ligand arm 1 substituent size and presence of cholesterol. In ligand-free, cholesterol-free CB_2_, the distribution of side-chain angles had a peak at ~ 280° (and very small peak at roughly 180o which will not be discussed further, since it is unlikely to relate to experimental results). In each case the average magnitude was increased when cholesterol was present (Fig. 9a). The difference in mean χ^1^ angles between cholesterol conditions for each bound ligand are shown in Fig. 9a and plotted in Fig. 9b. As the ligand size grew, the effect of cholesterol on the rotamer angle decreased.

**Figure 10:**
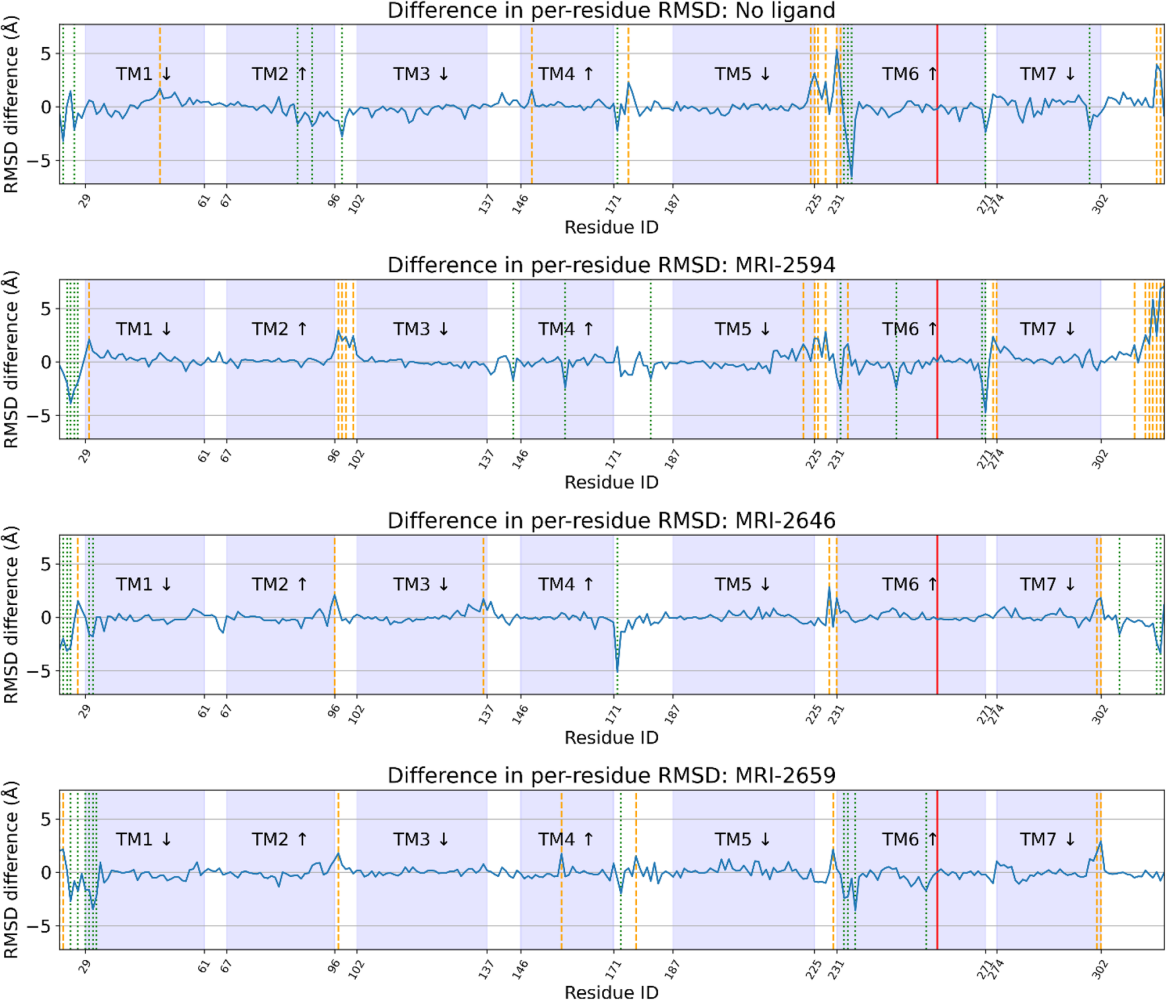
Difference in per-residue root-mean-square deviation (RMSD) between simulation and antagonist-bound cryo-EM structure. Trp258^6.48^ highlighted in red, residues with > 1 Å difference highlighted in orange (positive change) or green (negative change) dashed line. TM domains are annotated, with up and down arrows indicating direction of helix, with up from intracellular to extracellular.

Some amount of conformational change from the reference cryo-EM structure is to be expected in simulation, but we hypothesized that residues interfacing with G-protein, and therefore most likely to change the activation rate, would be preferentially affected as a function of the presence of cholesterol. To test this idea, we compared the average root-mean-square deviation (RMSD) of each Cα, with respect to the original cryo-EM structure at each simulation frame, between 0% cholesterol and 40% cholesterol conditions by subtraction. This comparison was made for free and each ligand-bound state. If a residue in the 40% cholesterol structure deviated more than the same residue in the 0% cholesterol structure, the resulting quantity would be positive, and if less, negative. Even though the reference cryo-EM structure is antagonist-bound, if there were no effect of cholesterol or ligand, the RMSD would be expected to be similar across conditions, resulting in zero RMSD difference. We found that residues that would interact with the G-protein complex, primarily in the ICL3 loop and the N-terminal side of TM6 (residues 222–236), were preferentially displaced in the presence of cholesterol across all ligand conditions (Fig. 10). The difference in average per-residue RMSD was relatively variable in this region compared to the rest of the structure, where this quantity was mostly close to zero (Fig. 10). The number of residues in this region with > 1 Å mean change in RMSD as a function of cholesterol presence was 9, 6, 2, and 3 in free, MRI-2594, MRI-2646, and MRI-2659-bound simulations respectively. This number decreased with ligand substituent size—though we cannot rule out other factors, particularly including the chemical composition of the substituent.

Given that RMSD change as a function of the presence of cholesterol was related to substituent size, we hypothesized that the root-mean-square fluctuation (RMSF) of each residue with respect to the average structure would also be affected in a systematic way (Supporting Fig. 8). A decrease in RMSF of specific residues would suggest a localized decrease in entropy. The ICL3 and first part of TM6 (residue 221–236) exhibited a relatively high RMSF (mean 2.58–3.80 Å with no cholesterol, 2.85–4.65 Å with 40% cholesterol). The magnitude of the RMSF was inversely correlated to the size of the ligand substituent. In unbound CB_2_, the RMSF in this region in the 40% cholesterol condition was substantially higher than in the 0% cholesterol condition (4.65 Å vs 2.58 Å). While relatively small in magnitude, the mean of absolute per-residue difference in RMSF as a function of the presence of cholesterol also decreased as ligand substituent size increased (1.01, 0.58, and 0.40 Å for MRI-2594, MRI-2646, and MRI-2659 respectively, shown in Supporting Fig. 8).

## Discussion

**Figure 11:**
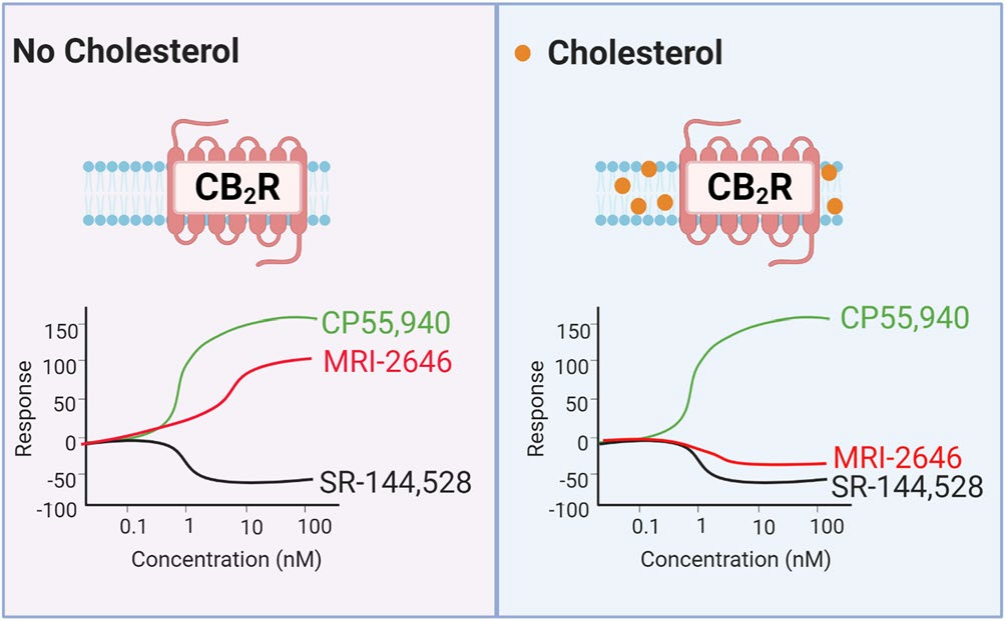
Membrane cholesterol dependent protean agonism.

Here we demonstrated that cholesterol increases the basal activation levels of CB_2_ receptor, thereby altering the pharmacological classification of novel synthetic cannabinoid ligands (Fig. 11). While in membranes devoid of cholesterol the MRI ligands act as partial agonist of CB_2_, in cholesterol-enriched membranes that same ligands became either inverse agonists or neutral antagonists of this receptor.

Some of us have previously reported the stabilizing effect of anionic lipids such as PG and PS on purified CB_2_ protein reconstituted in liposomes^43,47^. It was also shown that the negatively charged cholesterol derivative, cholesteryl hemisuccinate (CHS), stabilizes the recombinant CB_2_ protein in detergent micelles and liposomes^43^. At the same time, it was reported that cholesterol did not affect the activation of CB_2_ by the full agonist CP-55,940^47^. Since these observations were made on a CB_2_ receptor activated by the high affinity full agonist CP-55,940, the relatively moderate effects of the lipid matrix on receptor activation may have been masked.

Cholesterol is the major sterol found in higher eukaryotes^48^. The rigid planar structure of cholesterol modulates fluidity, thickness, curvature and permeability of membranes^49–51^. The presence of cholesterol in membranes increases ordering of lipid acyl chains. Physicochemical parameters of membranes have been implicated in the regulation of function of integral membrane proteins^49,52,53^. Lateral compression and hydrophobic matching between the lipid bilayer and transmembrane domains of the protein are affected by cholesterol content. These parameters are important for the structural stability of embedded membrane proteins^51^.

It was previously reported that cholesterol rafts in human immune cell membranes modulate the activity of CB_1_ receptor but do not affect the activation behavior of CB_2_ receptor^29^. That conclusion was reached by quantifying several signaling pathways in cells treated with methyl-β-cyclodextrin (MβCD). However, the effects of cholesterol/ MβCD were assessed in the presence of the full agonist of CB_2_, CP-55,940 that may have masked moderate effects on activation of CB_2_ receptor by the lipid bilayer. Here, by using a novel synthetic ligand MRI-2646 we demonstrated that the content of cholesterol in lipid bilayers modulates CB_2_ activation.

We have demonstrated that cholesterol increases the constitutive activity of the CB_2_ receptor. This effect was first shown in membrane preparations of several types of cells expressing CB_2_. One can argue that the difference between the membranes of mammalian cells (HEK, CHO) on the one hand, and insect and bacterial cells—on the other hand is not only in the content of cholesterol but in many other parameters including composition of phospholipids. Indeed, it has been reported that the content of phosphocholine (PC) lipids in HEK cell membranes is about 33% of total lipids, about two-fold higher compared to Sf9 membranes which have higher phosphoethanolamine (PE) (almost 40% of total lipids)^10^. The difference in lipid composition is even more pronounced when *E. coli* membranes are considered: they consist predominantly (almost 75%) of zwitterionic PE with the remainder consisting of anionic lipids (PG) and cardiolipin^54^. To prove that the presence or absence of cholesterol plays a major role in modulating the spontaneous signaling by CB_2_, we reconstituted the purified receptor into lipid bilayers of defined composition, containing POPC/POPG, 3/1, mol/mol and cholesterol in the range of 0–40% of total lipids. Indeed, the presence of cholesterol in these artificial bilayers resulted in an increase of spontaneous signaling by CB_2_. This effect was observed for the recombinant receptor isolated from *E. coli* cells as well as from the HEK Expi293F cells, providing additional evidence for an increase of basal activity of the receptor by cholesterol.

What are the mechanisms by which cholesterol modulates function of GPCR? There are several examples of GPCR that exhibit a certain affinity for cholesterol, and whose activities are regulated in response to the content of cholesterol in membranes. This includes the β2-adrenergic receptor and the μ-opioid receptor for which specific high affinity cholesterol binding sites have been reported near the transmembrane helices^7,45^.

A putative cholesterol binding sequence (CRAC) was reported for transmembrane helix 7 of human CB_1_ receptor^28^. This sequence was proposed to be involved in directing the interaction of CB_1_ receptor with cholesterol-rich microdomains of cell membranes. Moreover, the presence of a cholesterol molecule was recently reported in a crystal structure^55^ and a cryo-EM structure^56^ of CB_1_ receptor. At the same time, there was no evidence of a specific retention of cholesterol in a recently published CB_2_ crystal^31^ structure. There is cholesterol included in PDB 6PT0, a cryo-EM structure of CB_2_^44^, although its origin is unclear since the protein was expressed in Sf9 cells that produce very little if any cholesterol^10^. Also, any specific interaction of cholesterol with certain sites on the receptor may not explain why modulation of receptor function occurs at relatively high cholesterol concentrations in the lipid matrix surrounding the receptor.

Using MD simulations, we searched for structural and dynamical correlates of the experimental results. In simulations of free CB_2_, the ICL3-TM6 region, known to interact with G_α_, deviated significantly in RMSD/RMSF from the antagonist-bound conformation in the presence of cholesterol. Since the antagonist-bound conformation would be less likely to recruit G protein by definition, deviating from it is consistent with the large experimentally observed increase in constitutive activity. By contrast, the MRI-2659-bound structure showed relatively little deviation in this region, consistent with the strong antagonist activity of MRI-2659 in both cholesterol conditions.

The situation is less clear with the other two simulated ligands. We observed that the size of the ligand is inversely correlated with the effect of cholesterol on both Trp258 and ICL3-TM6. Yet this correlation does not map precisely to the corresponding changes in categorizing the ligands’ actions. When cholesterol is included in the membrane, the strong agonist MRI-2594 remains a (less-strong) agonist, the weaker agonist MRI-2646 becomes an inverse agonist, and the strong inverse agonist MRI-2659 remains as such (Fig. 3). The ligands are categorized relative to the baseline of ligand-free constitutive activity. The relative activity rank order of the MRI ligand series ligands (Fig. 3, y-axes) is preserved in both cholesterol conditions. We consider that the simulation-observed changes in the binding and ICL3-TM6 (i.e. G-protein binding) sites may be independent components contributing to the overall experimentally-observed effects, rather than ligand-specific effects that directly correlate with the pharmacological categories of ligands, since the simulation results correlated with the ligand sterics rather than their pharmacological categories. This is consistent with the hypothesis that the primary effect of cholesterol is to modify the baseline constitutive activity that defines how the tested ligands are categorized.

Our simulation cannot elucidate the allosteric pathway from binding pocket to G protein that would be responsible for the data—such an endeavor is well outside the scope of this work. While the chemical identity of the ligand is presumably important, we have not specifically addressed the effects of specific ligands beyond sterics. Future work might include simulating the CB_2_-G-protein complex and estimating the binding energy difference as a function of cholesterol and ligand, but this is an exceedingly large task.

The data do not explain how the chloro- substituent at the 6-position of the benzothiazole arm may regulate effects of cholesterol on CB_2_ activation. While the toggle switch Trp258^6.48^ functions as an important molecular determinant in activation or deactivation of the receptor, it sheds limited light on differential interactions leading to neutral vs. inverse agonism. That the compound MRI-2654 with a bromo-substituent still behaves as an inverse agonist attests to the subtle difference in size and electronegativity of the chloro-group in influencing the molecular dynamics and signaling processes resulting in modulation of receptor function.

By using MRI-2646 ligand we demonstrate that cholesterol increases the constitutive activity of CB_2_ receptor. The content of cholesterol in preparations of cell membranes expressing CB_2_ correlates with an increase in basal signaling through CB_2_, which could be reversed by depletion of cholesterol using cyclodextrin. These results suggest that the pharmacological properties of synthetic ligands can be influenced by the cholesterol composition of cell membranes harboring cannabinoid receptor. Such a regulatory mechanism may contribute for well documented tissue- and cell-specific differences in the efficacy of partial CB_2_ agonists, such as the endocannabinoid anandamide or the plant-derived cannabinoid Δ^9^-tetrahydrocannabinol^57^. For example, THC acted as a full CB_2_ agonist in suppressing interferon-γ-induced activation of microglia^58^, whereas it had no CB_2_ agonist activity and acted as a CB_2_ antagonist by blocking 2-AG-induced migration of natural killer cells^59^, which have high levels of membrane lipid, including cholesterol^60^.

## Methods

### Materials

Chromatographic resin Ni–NTA was purchased from Qiagen (Germantown, MD). Streptactin XT was from IBA Life Sciences (Goettingen, Germany). The detergents CHAPS (3-[(3-Cholamidopropyl)-Dimethylammonio]-1-Propane Sulfonate] • N,N-Dimethyl-3-Sulfo-N-[3-[[3α,5β,7α,12α)-3,7,12-Trihydroxy-24-Oxocholan-24-yl]Amino]propyl]-1-Propanaminium Hydroxide, Inner Salt) , LMNG (Lauryl Maltose Neopentyl Glycol) and DDM (Dodecyl-β-D-Maltoside) were from Anatrace (Maumwee, OH). CHS-Tris salt was from Anatrace. The detergent Façade-TEG (3a,7a,12a-tri-((O-b-D-glucopyranosyl)ethyloxy)-cholane) and lipids POPC, POPG, brain lipid extract and cholesterol were from Avanti Polar Lipids (Alabaster, AL).

The potent non-selective CB_2_ agonist CP-55,940 ((-)-*cis*-3[2-hydroxy-4-(1,1-dimethylheptyl)phenyl]-*trans*-4-(3-hydroxtpropyl) cyclohexanol, the high affinity selective CB_2_ inverse agonist SR-144,528 5-(4-Chloro-3-methylphenyl)-1-[(4-methylphenyl)methyl]-N-[(1S,2S,4R)-1,3,3-trimethylbicyclo[2.2.1]hept-2-yl]-1H-pyrazole-3-carboxamide were from Cayman Chemical (Ann Arbor, MI). ^3^H-labeled CP-55,940 was from Perkin Elmer Life Sciences (Akron, OH). All other reagents were from Sigma-Aldrich (USA).

Preparations of CHO cell membranes expressing CB_2_ were obtained from the following sources: PerkinElmer (Cat. No ES111-M400UA, Billerica, MA), Millipore ChemiScreen (Cat. No P34972, Burlington, MA) and Applied Cell Sciences (Cat. No A318, Rockville, MD). Preparations of Sf9 cell membranes expressing CB_2_ were from Signal Screen (Cat. No 6110130, Rockville, MD).

Expi293F HEK cells were obtained from ThermoFisher Scientific (Cat. No: A14527), and CB_2_ expressed and membranes obtained in house as described elsewhere^42^. The *E. coli* cells BL21 (DE3) were obtained from EMD Millipore-Sigma (Cat. No. 69450), and CB_2_ expressed and membranes were obtained in house as described elsewhere.^34,61^.

### Chemistry

Commercially available regents were purchased and used as is. Proton (^1^H NMR) spectra were recorded on a Varian 400 or Bruker 500 MHz spectrometer in solvents indicated with the values given in ppm (TMS as internal standard) and *J* (Hz) assignments of proton resonance coupling. Mass spectra (HRMS) were recorded on a JEOL SX102a mass spectrometer. Thin layer chromatography (TLC) analyses were carried out on 5 cm × 10 cm silica gel GHLF 0.25 mm plates using various gradients of EtOAc:*n*-hexane with visualization under UV light. Flash column chromatography was performed on Combiflash system. Product yields are reported as un-optimized. Study compounds had ≥ 95% purity. Purity and structural characterization was done by a combination of TLC, ^1^H-NMR, and LC/MS. LC–MS detection was carried out on Agilent 1200 using two different methods/columns: Luna C_18_ 3 um (3 × 75 mm) where the mobile phase was 4% to 100% acetonitrile (0.05% TFA) standard gradient and EC18, 2.7 um (3 × 50 mm) where the method was 50% acetonitrile in water (0.1% formic acid) for 3 min ramping up to 98% acetonitrile over 7.5 min. The LC–MS chromatogram showed the correct molecular (MH^+^) ion as well as a single peak at UV (254 nm).

### Synthesis and characterization of MRI-2687^28^ and MRI-2594^31,32,62^

#### Synthesis and characterization of MRI-2646, MRI-2654, MRI-2653 and MRI-2659 were carried as outlined in Li et al.^28^

*N*-(6-methyl-3-(2-hydroxyethyl)benzo[*d*]thiazol-2(3*H*)-ylidene)-2,2,3,3-tetramethylcyclopropane-1-carboxamide (**4a**)^28^.

*N*-(6-chloro-3-(2-hydroxyethyl)benzo[*d*]thiazol-2(3*H*)-ylidene)-2,2,3,3-tetramethylcyclopropane-1-carboxamide (**4b**).
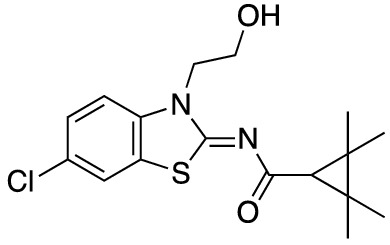


2-amino-6-chlorobenzothiazole (1.84 g, 10.0 mmol) gave compound **4b** (700 mg, 20%) as a white powder over two steps. Mp 147–149 °C;^1^H-NMR (400 MHz, CDCl_3_): δ 7.56 (s, 1H), 7.36 (d, *J* = 8.7 Hz, 1H), 7.22 (d, *J* = 8.7 Hz, 1H), 4.49 (t, *J* = 4.6 Hz, 3H), 4.06 (t, *J* = 4.6 Hz, 3H), 1.58 (s, 2H), 1.32 (s, 8H), 1.22 (s, 8H). LCMS [M + H]^+^: 353.2.

*N*-(6-bromo-3-(2-hydroxyethyl)benzo[*d*]thiazol-2(3*H*)-ylidene)-2,2,3,3-tetramethylcyclopropane-1-carboxamide (**4c**).
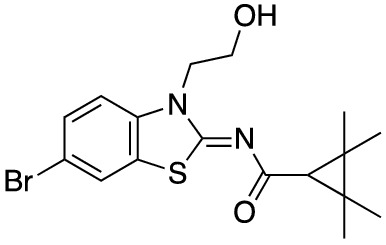


2-amino-6-bromobenothiazole (2.29 g, 10.0 mmol) gave compound **4c** (950 mg, 24%) as a white powder over two steps. Mp 173–175 °C;^1^H-NMR (400 MHz, CDCl_3_): δ 7.70 (s, 1H), 7.50 (d, *J* = 8.6 Hz, 1H), 7.17 (d, *J* = 8.7 Hz, 1H), 4.49 (t, *J* = 4.5 Hz, 2H), 4.17 (s, 1H), 4.06 (s, 2H), 1.58 (s, 1H), 1.32 (s, 6H), 1.22 (s, 6H). LCMS [M + H]^+^: 397.1.

*N*-(3-(2-hydroxyethyl)-6-methoxybenzo[*d*]thiazol-2(3*H*)-ylidene)-2,2,3,3-tetramethylcyclopropane-1-carboxamide (**4d**).
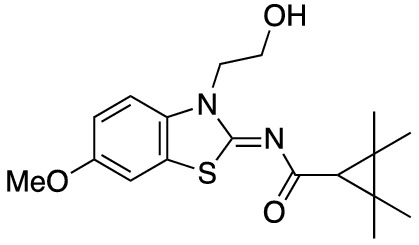


2-amino-6-methoxybenothiazole (1.8 g, 10.0 mmol) gave compound **4d** (920 mg, 26%) as a white powder over two steps. Mp 164–166 °C;^**1**^**H-NMR** (400 MHz, CDCl_3_): δ 7.18 (d, *J* = 8.9 Hz, 1H), 7.11 (s, 1H), 6.99–6.96 (m, 1H), 4.82 (s, 1H), 4.48 (t, *J* = 4.5 Hz, 2H), 4.05 (d, *J* = 3.5 Hz, 2H), 3.83 (s, 3H), 1.55 (s, 4H), 1.32 (s, 6H), 1.21 (s, 6H). LCMS [M + H]^+^: 349.2.

*N*-(3-(2-hydroxyethyl)-6-(trifluoromethoxy)benzo[*d*]thiazol-2(3*H*)-ylidene)-2,2,3,3-tetramethylcyclopropane-1-carboxamide (**4e**).
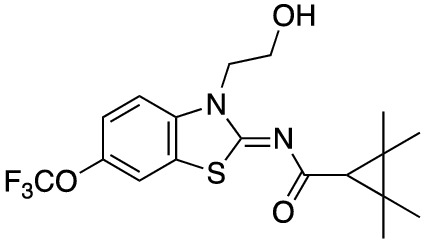


2-amino-6-trifluoromethoxybenothiazole (1.5 g, 6.4 mmol) gave compound **4** (610 mg, 24%) as a white powder over two steps. Mp 149–151 °C; ^**1**^**H-NMR** (400 MHz, CDCl_3_): δ 7.46 (s, 1H), 7.30 (t, *J* = 7.6 Hz, 2H), 4.51 (s, 2H), 4.07 (s, 3H), 1.59 (s, 1H), 1.32 (s, 6H), 1.22 (s, 6H). LCMS [M + H]^+^: 403.2.

2-(6-methyl-2-((2,2,3,3-tetramethylcyclopropane-1-carbonyl)imino)benzo[*d*]thiazol-3(2*H*)-yl)ethyl acetate (**MRI-2687**) (**2a**)^28^.

2-(6-Chloro-2-((2,2,3,3-tetramethylcyclopropane-1-carbonyl)imino)benzo[*d*]thiazol-3(2*H*)-yl)ethyl acetate (**MRI-2646**) (**2b**).
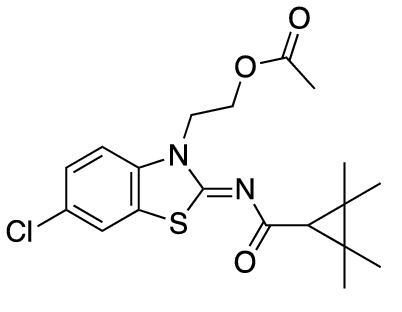


Compound **4b** (300 mg, 0.85 mmol) was used as a starting material to give compound **2b** (195 mg, 58%) as a white powder. Mp 123–125 °C;^1^H-NMR (400 MHz, CDCl_3_): δ 7.54 (s, 1H), 7.34 (d, *J* = 8.8 Hz, 1H), 7.23 (s, 1H), 4.56 (t, *J* = 5.2 Hz, 2H), 4.46 (t, *J* = 5.3 Hz, 2H), 1.95 (s, 3H), 1.64 (s, 1H), 1.33 (s, 6H), 1.23 (s, 6H). LCMS [M + H]^+^: 395.2.

2-(6-Bromo-2-((2,2,3,3-tetramethylcyclopropane-1-carbonyl)imino)benzo[*d*]thiazol-3(2*H*)-yl)ethyl acetate (**MRI-2654**) (**2c**).
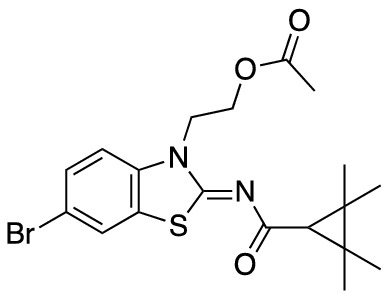


Compound **4c** (300 mg, 0.75 mmol) was used as a starting material to give compound **2c** (140 mg, 42%) as a white powder. Mp 127–129 °C;^1^H-NMR (400 MHz, CDCl_3_): δ 7.68 (s, 1H), 7.48 (d, *J* = 8.3 Hz, 1H), 7.19 (d, *J* = 8.6 Hz, 1H), 4.55 (d, *J* = 5.3 Hz, 2H), 4.47 (d, *J* = 5.2 Hz, 2H), 1.95 (s, 3H), 1.64 (s, 1H), 1.57–1.49 (m, 7H), 1.33 (s, 6H), 1.23 (s, 6H). LCMS [M + H]^+^: 439.2.

2-(6-Methoxy-2-((2,2,3,3-tetramethylcyclopropane-1-carbonyl)imino)benzo[*d*]thiazol-3(2*H*)-yl)ethyl acetate (**MRI-2653**) (**2d**).
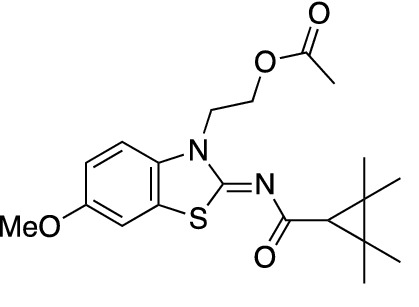


Compound **4d** (400 mg, 1.1 mmol) was used as a starting material to give compound **2d** (230 mg, 51%) as a white powder. Mp 154–156 °C;^1^H-NMR (400 MHz, CDCl_3_): δ 7.21 (s, 1H), 7.09 (s, 1H), 6.96 (d, *J* = 9.3 Hz, 1H), 4.55 (d, *J* = 5.3 Hz, 2H), 4.47 (d, *J* = 5.2 Hz, 2H), 7.24–1.22 (m, 82H), 3.83 (s, 3H), 1.95 (s, 3H), 1.62 (s, 1H), 1.34 (s, 6H), 1.22 (s, 6H). LCMS [M + H]^+^: 391.2.

2-(2-((2,2,3,3-tetramethylcyclopropane-1-carbonyl)imino)-6-(trifluoromethoxy)benzo[*d*]thiazol-3(2*H*)-yl)ethyl acetate (**MRI-2659**) (**2e**).
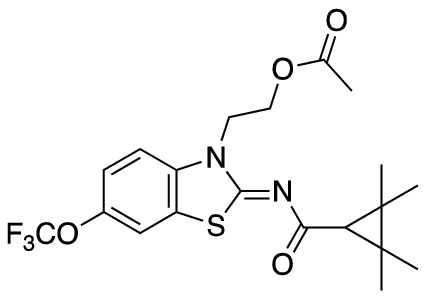


Compound **4e** (300 mg, 0.75 mmol) was used as a starting material to give compound **2e** (190 mg, 57%) as a white powder. Mp 81–83 °C; ^1^H-NMR (400 MHz, CDCl_3_): δ 7.44 (s, 1H), 7.32 (d, *J* = 8.8 Hz, 1H), 7.26 (s, 2H), 4.57 (d, *J* = 5.4 Hz, 2H), 4.47 (t, *J* = 5.4 Hz, 2H), 1.95 (s, 3H), 1.64 (s, 1H), 1.33 (s, 6H), 1.23 (s, 6H). LCMS [M + H]^+^: 445.3.

### ***CB***_***2***_*** expression in E. coli and purification***

CB_2_ was expressed as a fusion with the maltose binding protein (MBP) in BL21 (DE3) *E. coli* culture and purified on milligram-scale as previously described^41,63^. In brief, 10 L of 2xYT media containing 0.2% glucose supplemented with ampicillin was inoculated with an overnight culture of *E. coli*. After reaching an optical density of 0.4, CB_2_ expression was induced by addition of 0.5 mM IPTG and 2.5 μM CB_2_ agonist CP-55,490. Expression was conducted for additional 42 h at 20 °C. After expression, cells were harvested by centrifugation, washed with cold PBS, and lysed in a cell homogenizer (Avestin). Receptor was solubilized for 1 h at 4 °C under continuous stirring by addition of concentrated detergent to final concentrations of (0.1% CHS, 1.0% DDM, 0.5% CHAPS, all w/v). The insoluble material was removed by centrifugation at 170,000 × *g* for 1 h and the solubilized receptor was then purified by the affinity chromatography in 50 mM Tris pH 7.5, 150 mM NaCl, 0.1% CHS, 0.1% DDM, 0.5% CHAPS, all w/v; 30% glycerol (v/v) and 10 μM CP-55,490 (buffer A) on Ni–NTA (Qiagen). MBP fusion partner was then removed upon incubation with the tobacco etch virus (TEV) protease for 4 h at 4 °C, and the released receptor was further purified by chromatography on StrepTactin XT (IBA Biosciences) and eluted in buffer A supplemented with 50 mM biotin as described previously^41^.

### ***CB***_***2***_*** expression in HEK cells and purification***

Biomass from 3 L of Expi293F GNTI^-^ cells expressing CB_2_ containing N-terminal twin-Streptag and C-terminal His_10_ tag was obtained according to manufacturer’s protocol (ThermoFisher Scientific). Protein was solubilized in detergents and purified by the two successive rounds of affinity chromatography on Ni–NTA resin and StrepTactin XT resin as described^41^.

### ***Removal of the CHS and ligand from purified CB***_***2***_

An amount of 2 mg of purified CB_2_ was bound to 1200 μL of HisPur Co^2+^ (ThermoFisher) resin in buffer A and incubated under shaking for 2 h at 4 °C. The protein sample was then transferred to a disposable gravity column and washed with 40 column volumes (CV) of 0.5% CHAPS/0.1% DDM in 50 mM Tris–HCl, pH 7.5, 150 mM NaCl. Protein was then eluted with 6xCV of the same buffer supplemented with 250 mM imidazole; combined eluates concentrated on 30 kDa MWCO spin concentrator and washed 3 times to remove imidazole. Concentrated protein was supplemented with 15% glycerol and aliquots stored at − 80 °C until further use. To confirm that both ligand and CHS have been removed from the sample, 20 μL aliquot was mixed with 300 μL of chloroform–methanol mixture (1:1 v/v) and ^1^H-NMR spectra acquired.

### ***Reconstitution of CB***_***2***_*** into liposomes***

Reconstitution of the purified CB_2_ into liposomes was performed as described earlier^43^. Briefly, 200 μg of the purified protein was mixed with 2 mg of lipid mixture (POPC:POPG, 3:1, mol/mol without or with addition or 20 mol% or 40 mol% of cholesterol) solubilized in 1% CHAPS at a concentration of 5 mg lipid/mL, and incubated on ice for 30 min. The detergents were then removed on 4 mL Detergent Removal spin column (Pierce), according to manufacturer’s instructions. The combined filtrate containing proteoliposomes was collected, and aliquots frozen in liquid nitrogen. Frozen liposomes were stored at − 80 °C until further use. Content of protein in proteoliposomes was determined by BioRad DC assay.

### ***Ligand-binding assay in hCB***_***2***_***-CHO-K1 cell membranes***

The assay was performed as described previously^64^. Briefly, binding affinity of the compounds to CB_2_R was determined by radioligand displacement assays using 0.2 nM of [^3^H] CP-55,940 as the radioligand. Plasma membranes were from cultured CHO-K1 cells stably transfected with human CB_2_R (Perkin Elmer). Two microgram plasma membrane protein was used in a 1 mL reaction mixture. Ki values were derived by computerized curve fitting and using the Cheng-Prusoff equation to account for the affinity of the radioligand, using the GraphPad Prism 8 program (GraphPad Prism Software Inc.).

### ***[***^***35***^***S] GTPγS binding assay in hCB***_***2***_***-CHO-K1 cell membranes***^65^

[^35^S] GTPγS binding was assayed as described earlier^65^ with slight modifications. Briefly, hCB_2_-CHO-K1 cell membranes (4 µg) were incubated with 0.05 nM [^35^S] GTP*γ*S, and the indicated concentrations of ligands in TEM buffer (50 mM Tris–HCl, 0.2 mM EGTA, and 9 mM MgCl_2_, pH 7.4) containing 100 µM GDP, 150 mM NaCl, and 0.1% (w/v) bovine serum albumin in a total volume of 1 ml for 60 min at 30 °C.

### ***[***^***35***^***S] GTP nucleotide exchange (GEF) assays***

The subunits of G protein were expressed and purified as described previously^63^. The nucleotide exchange assay was performed as previously described^66^.

### Molecular dynamics simulations

The CHARMM36 force field^67^ was used. Before ligand parameterization, each ligand was geometry optimized using the B3LYP/6-31G** quantum mechanics level of theory and basis set using Gaussian09^68^. Ligand parameters were derived from CGenFF^69^; these high affinity ligands were not expected to explore the extremes of their conformational space.

The CB_2_ structure starting point was the cryo-EM structure previously described (Protein Data Bank: 6PT0). This was oriented and placed in a lipid membrane using the Orientations of Proteins in Membranes (OPM) database^70^ using the CHARMM-GUI^71^ input generator. In the with-cholesterol condition, the lipid membrane consisted of 40% cholesterol, and POPC:POPG in a 3:1 ratio. Sodium and chloride atoms were added to 0.15 M with excess for electroneutrality. The cholesterol molecules present in the cryo-EM structure were retained, particularly since the extracellular cholesterol conformations were analogous to those observed in the structurally similar mu opioid receptor. The system was minimized and equilibrated with side chain and backbone restraints which were subsequently released, and production simulations were run in the isothermic-isobaric ensemble at 303.15 K using NAMD 2.13 with GPU extensions. Particle Mesh Ewald summation of long-range interactions was used, as were the Langevin barostat and thermostat.

## Supplementary Information

Supplementary Information

## Abbreviations

GPCR: G protein-coupled receptor
ICL: Intracellular loop (in GPCR)
TM: Transmembrane domain (in GPCR)
HEK293: Human embryonic kidney
CHO: Chinese hamster ovary
*E. coli*: *Escherichia coli*
Sf9 cells: A clonal isolate of *Spodoptera frugiperda* baculovirus infected insect cell
MD: Molecular dynamics
hCB_2_: Human cannabinoid receptor CB_2_
hCB_1_: Human cannabinoid receptor CB_1_
MBP: Maltose binding protein of *E. coli*
CP-55,940: Synthetic cannabinoid agonist 2-[(1R,2R,5R)-5-Hydroxy-2-(3-hydroxypropyl)cyclohexyl]-5-(2-methyloctan-2-yl)phenol
[^3^H]-CP55,940: Radiolabeled synthetic ligand CP-55,940
SR-144,528: Synthetic cannabinoid inverse agonist 5-(4-Chloro-3-methylphenyl)-1-[(4-methylphenyl)methyl]-N-[(1S,2S,4R)-1,3,3-trimethylbicyclo[2.2.1]heptan-2-yl]-1H-pyrazole-3-carboxamide
[^35^S]-GTPγS: Radiolabeled synthetic analog of guanosine triphosphate
MβCD: Methyl-β-cyclodextrin
G_α__i1_ and G_β__1__γ__2_: Subunits of heterotrimeric G protein
GEF assay: Determines rates of nucleotide exchange on the G_α_ subunit of G protein
POPC: 1-Palmitoyl-2-oleoyl-glycero-3-phosphocholine
POPG: 1-Palmitoyl-2-oleoyl-sn-glycero-3-phospho-(1′-rac-glycerol)
CHS: Cholesteryl hemisuccinate
RMSD: Root-mean-square deviation
RMSF: Root mean square fluctuation
