# Supplementary material for: Cholesterol as a modulator of cannabinoid receptor CB2 signaling
Source: Sci Rep. 2021 Feb 12;11:3706. doi: 10.1038/s41598-021-83245-6 (PMC7881127; doi:10.1038/s41598-021-83245-6)
Supplement: Supplementary file 1 — Supplementary Information [file 41598_2021_83245_MOESM1_ESM.docx]

**Cholesterol as a modulator of cannabinoid receptor CB_2_ signaling**

**Alexei Yeliseev^1^*, Malliga R. Iyer^1^*, Thomas T. Joseph^2^, Nathan J. Coffey^1^, Resat Cinar^1^, Lioudmila Zoubak^1^, George Kunos^1^, Klaus Gawrisch^1^**

From the **^1^**National Institute on Alcohol Abuse and Alcoholism, National Institutes of Health, Bethesda, MD 20852, USA and **^2^**Department of Anesthesiology and Critical Care, Perelman School of Medicine, University of Pennsylvania, Philadelphia, PA 19104

*To whom correspondence should be addressed:

Alexei A. Yeliseev: [yeliseeva@mail.nih.gov](mailto:yeliseeva@mail.nih.gov);

Malliga R. Iyer: [malliga.iyer@nih.gov](mailto:malliga.iyer@nih.gov)

**Supplementary data**


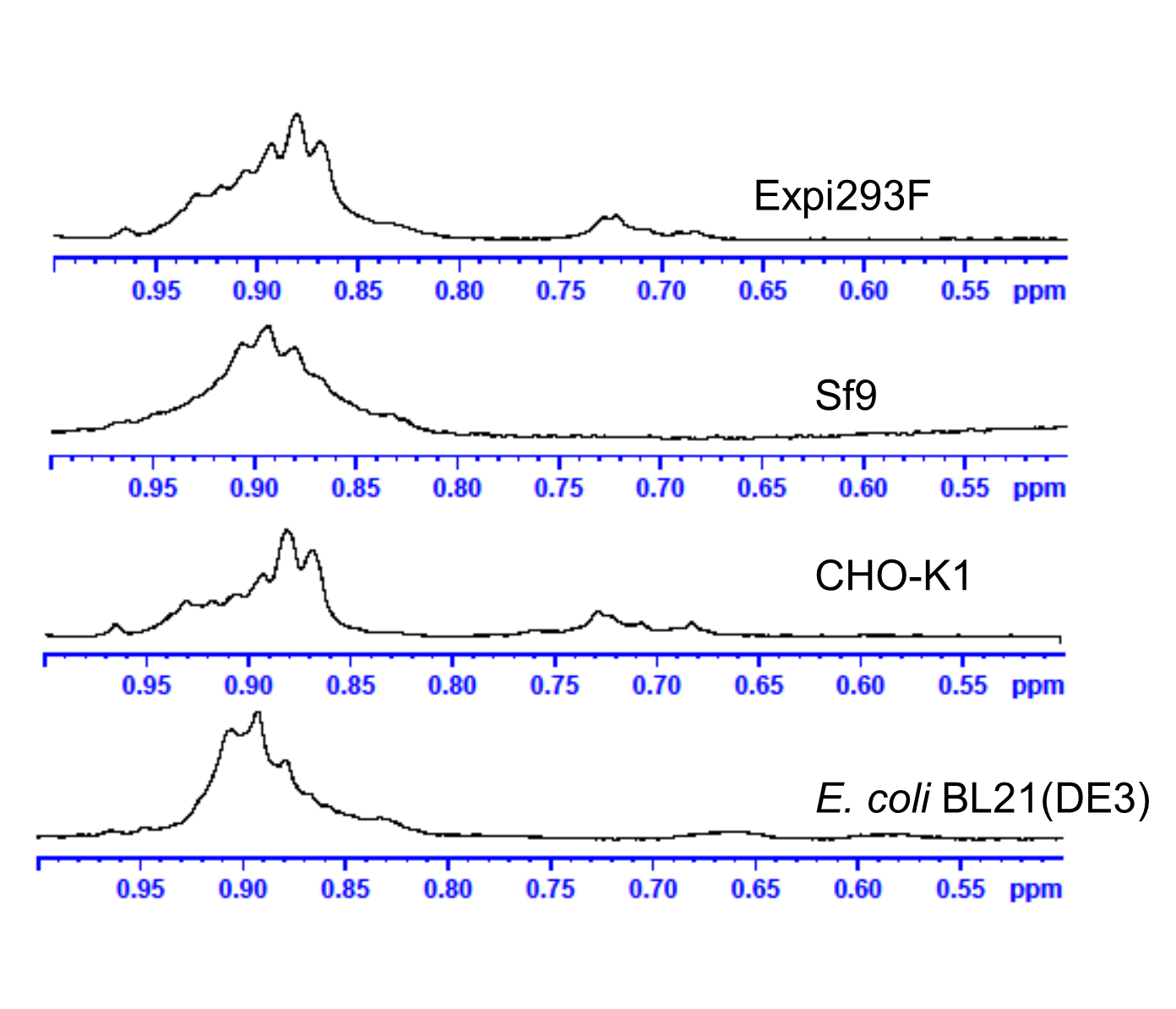


**Supplementary Figure 1.** ^1^H-NMR analysis of lipid composition of membrane preparations from cells expressing CB_2_. The lipids were extracted from membranes by the Folch method, and content of lipids was determined by ^1^H-NMR. The band of resonances from 1- 0.8 ppm originates from terminal methyl groups of hydrocarbon chains while the band from 0.75 – 0.65 ppm is characteristic for the C_18_ methyl resonances of cholesterol and other sterols with a gonane backbone methylated at the C_13_ carbon. Concentration of cholesterol is expected to dominate spectral intensity. The *E. coli* lipid extract displays two broad resonances of unknown origin, centered at 0.66 ppm and 0.58 ppm that are absent in the other membrane extracts. Integration of lipid methyl- and stearyl C_18_ resonance bands yields the cholesterol content in lipid extracts in mol% of total phospholipids: *E. coli* - 0%, CHO-K1- 30 %, SF9 - 0%, Expi293F^TM^-19 %. ^1^H-NMR spectra were recorded at 800 MHz, a relaxation delay of 3 s and 10,240 scans.

| **CB_2_ membrane preparation** | **Density of ligand binding sites, Bmax, pmol/mg protein** |
| --- | --- |
| CB2 in *E. coli* membranes | 29.3 |
| Sf9 (Signal Screen) | 22.1 |
| CHO (Perkin Elmer) | 34* |
| CHO (Applied Cell Sciences) | 29.3 |
| CHO (EMD Millipore) | 50* |
| HEK Expi293F | 23.2 |

**Supplementary Figure 2. ^3^H-CP-55,940 saturation binding on membrane preparations expressing CB_2_ receptor.** Ligand binding experiments were performed in triplicates as described in Methods, and data analyzed using GraphPad Prism 8. 1-5 μg of membrane protein per each concentration points. *Indicates values taken from material data sheet provided by the vendor.

**a**


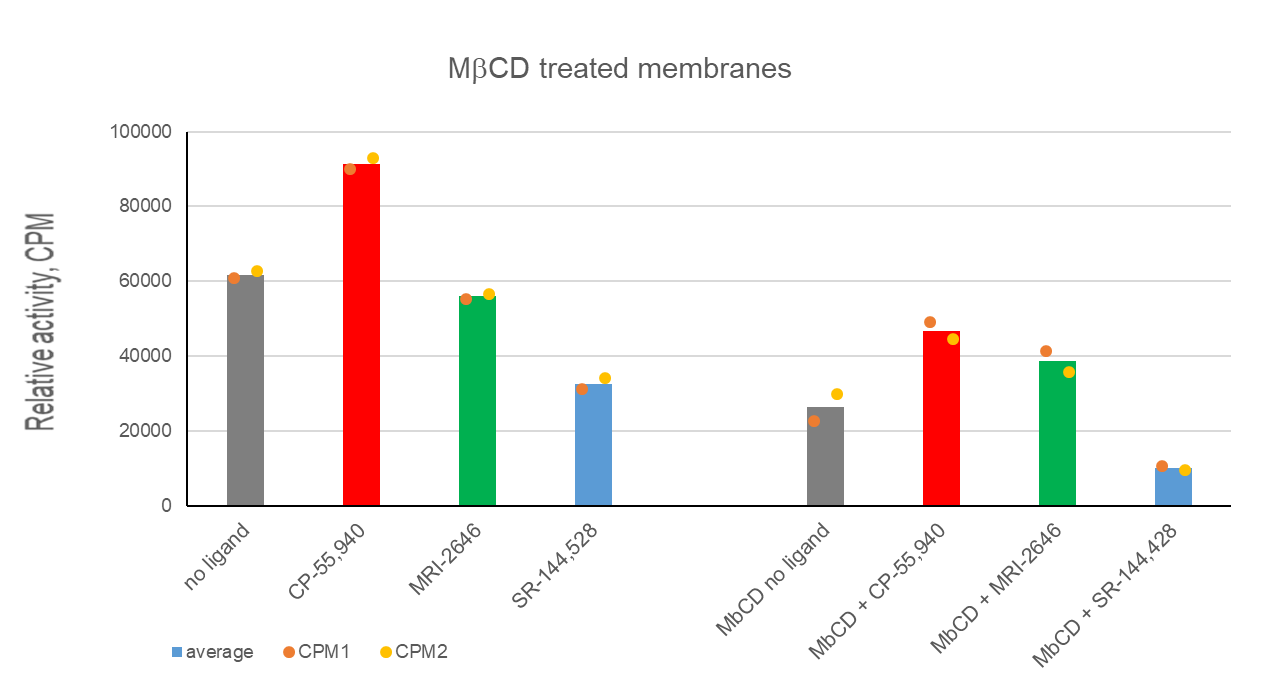


**b**

**
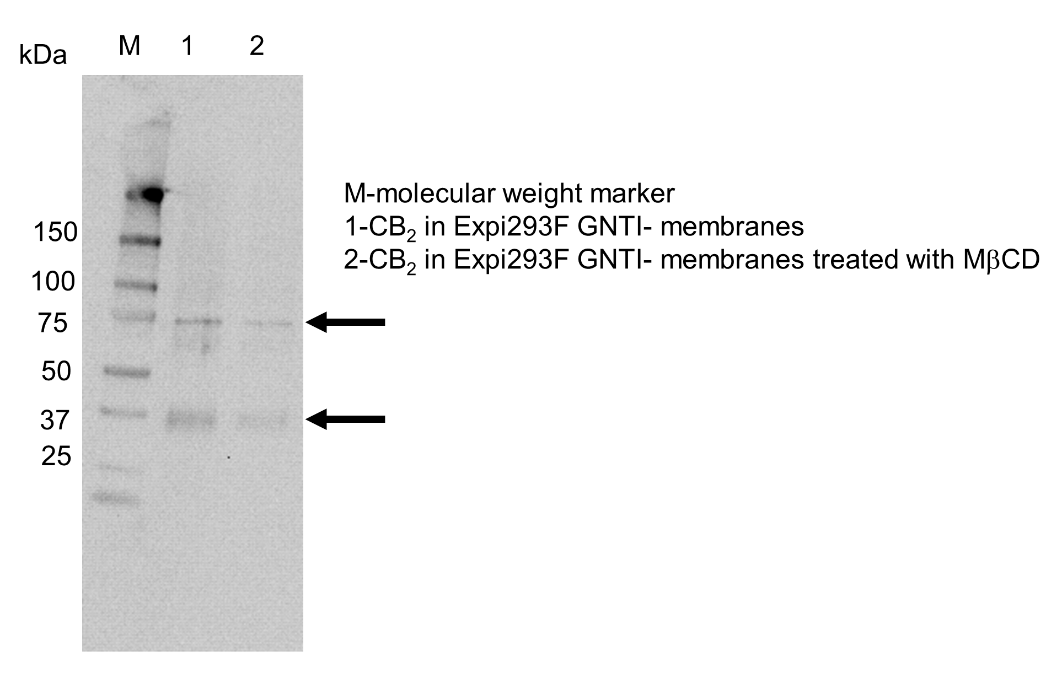
**

**Supplementary Figure 3. a, G protein activation on Expi293F GNTI^-^ membranes expressing CB_2_, untreated- or treated with MβCD; b, Western blot analysis of expression levels of receptor in membranes.** Where indicated, membranes were treated with 20 mM MβCD for 1 h, washed with PBS, and G protein activation measured as described in Methods. Bars represent an average of two independent measurements with individual data points shown by dots; b, 10 μg of membrane protein per lane. Blot were probed with antibodies against CB_2_ (ThermoFisher Scientific). Arrows indicate monomer and dimer (formed at conditions of SDS-PAGE) of CB_2_. There was no immunoreactive band in membrane preparations of Expi293FTM GNTI- not expressing the receptor (not shown).

**a**

| **CB_2_ membrane preparation** | **Density of ligand binding sites, Bmax, pmol/mg protein** |
| --- | --- |
| CB2 in *E. coli* membranes | 23.3 |
| Membranes treated with MβCD | 20.1 |

**b**

**
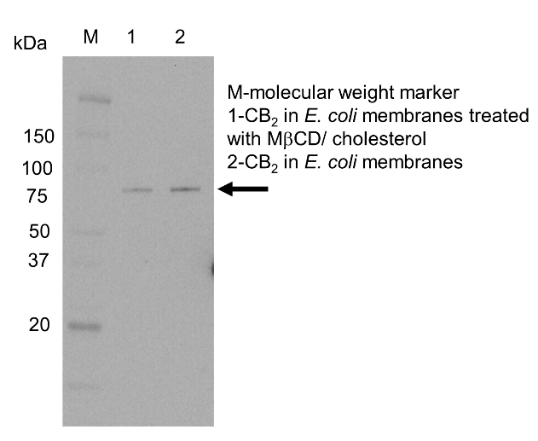
**

**Supplementary Figure 4. (a) ^3^H-CP-55,940 saturation binding; and (b) Western blot analysis of membrane preparations expressing CB_2_ receptor treated with MβCD/cholesterol.** Ligand binding experiments were performed in triplicates as described in Methods, and data analyzed using GraphPad Prism 8. 1-5 μg of membrane protein per each concentration point. **b,** Western blot analysis of MBP-CB_2_ fusion protein in membrane preparations: 10 μg of membrane protein per lane. Blots were probed with antibodies against CB_2_ (ThermoFisher Scientific). Arrow indicates position of the fusion protein MBP-CB_2_. There was no immunoreactive band in the control membrane preparation of *E. coli* cells not expressing the receptor (not shown).

**a**

**
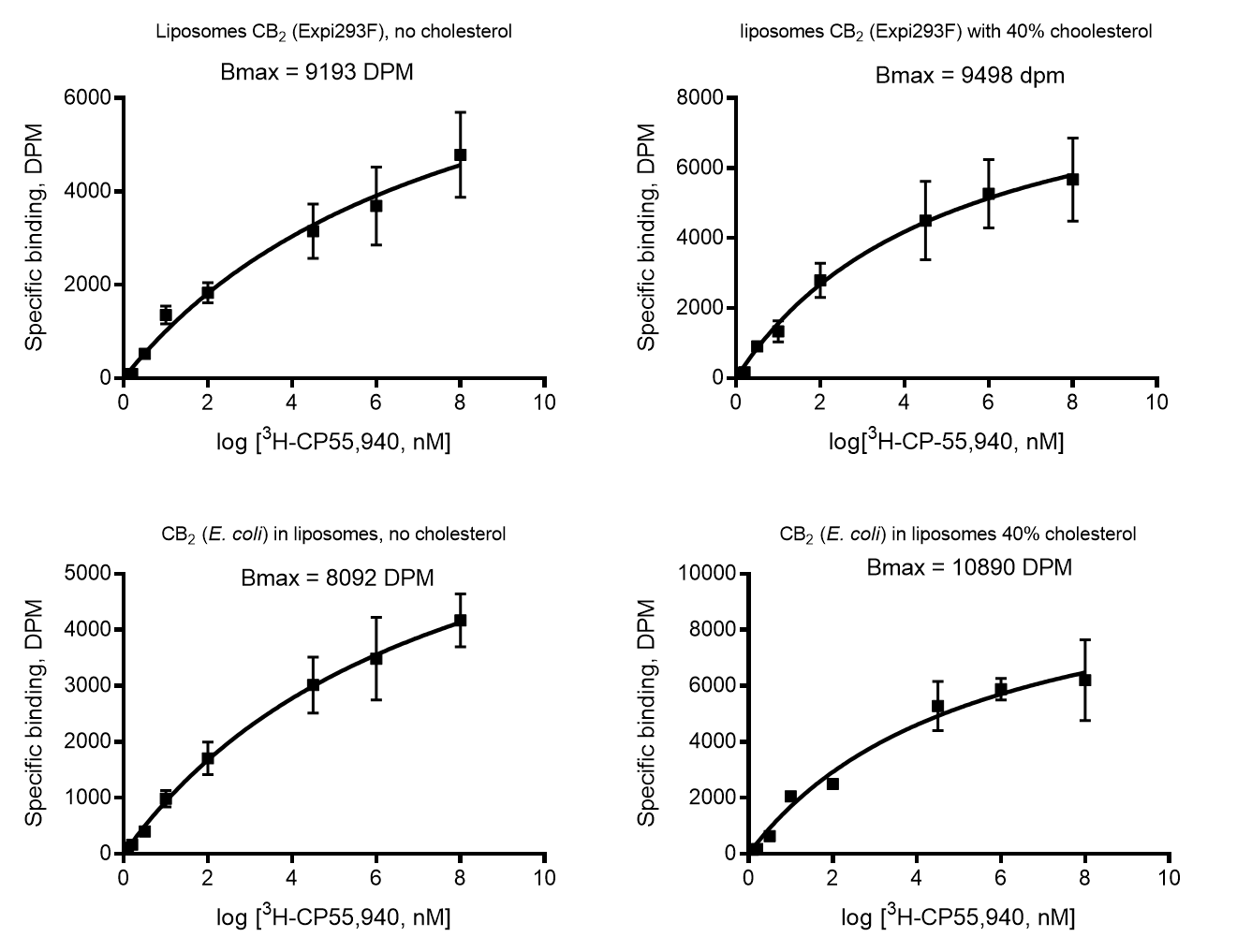
**

**b**

**
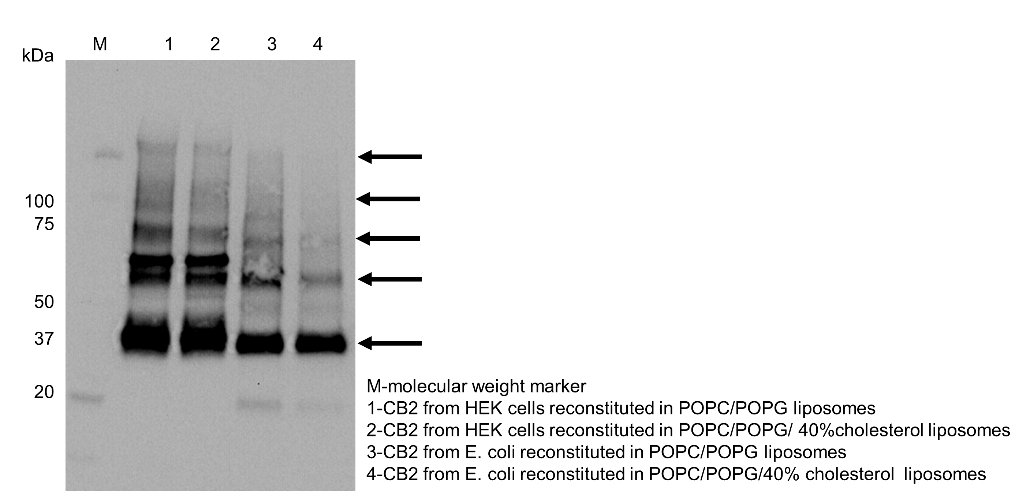
**

**Supplementary Figure 5. (a) ^3^H-CP-55,940 saturation binding; and (b) Western blot analysis of membrane preparations expressing CB_2_ receptor.** Ligand binding experiments were performed in triplicates as described in Methods, and data analyzed using GraphPad Prism 8. 25 ng of liposome protein sample per each concentration point. Western blot analysis of CB_2_ receptor in membrane preparations: ~50 ng of membrane protein per lane. Blot was probed with antibodies against CB_2_ (ThermoFisher Scientific). Arrows indicate likely positions of CB_2_ monomer, dimer, and higher oligomers (formed at conditions of SDS-PAGE).


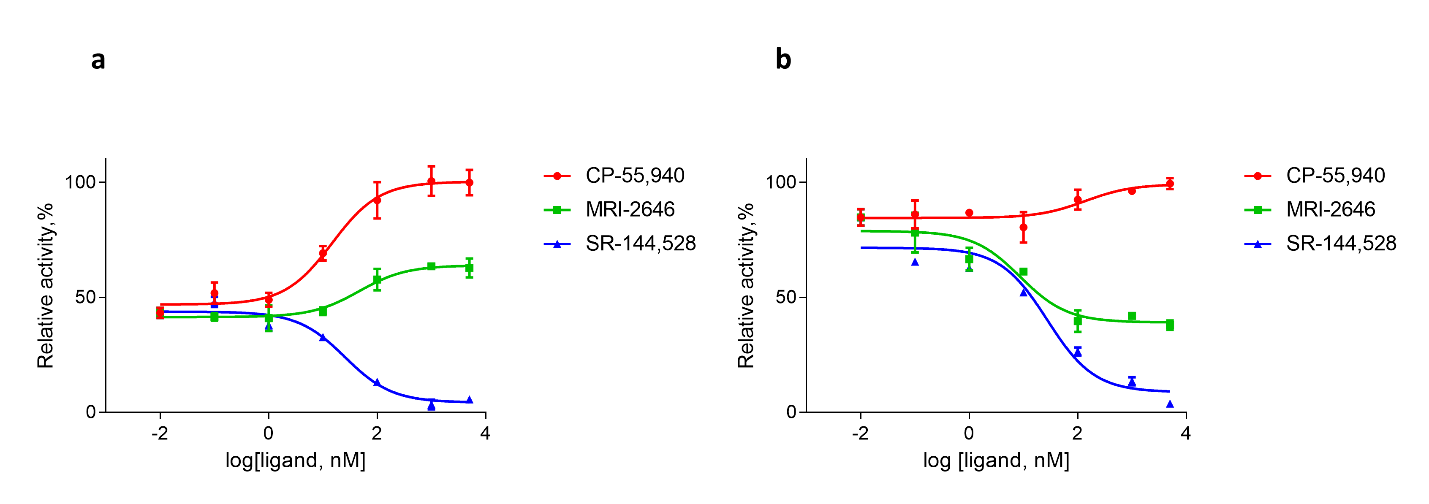


**Supporting Figure 6. GEF on CB_2_, purified from Expi293F GNTI^-^ cells and reconstituted into liposomes of a defined composition. a,** liposomes contain POPC/POPG, 3/1, mol/mol; b, liposomes contain POPC/POPG, 3/1, mol/mol/ plus 20 mol% cholesterol, relative to total phospholipids. Each data point represents an average of four independent measurements.


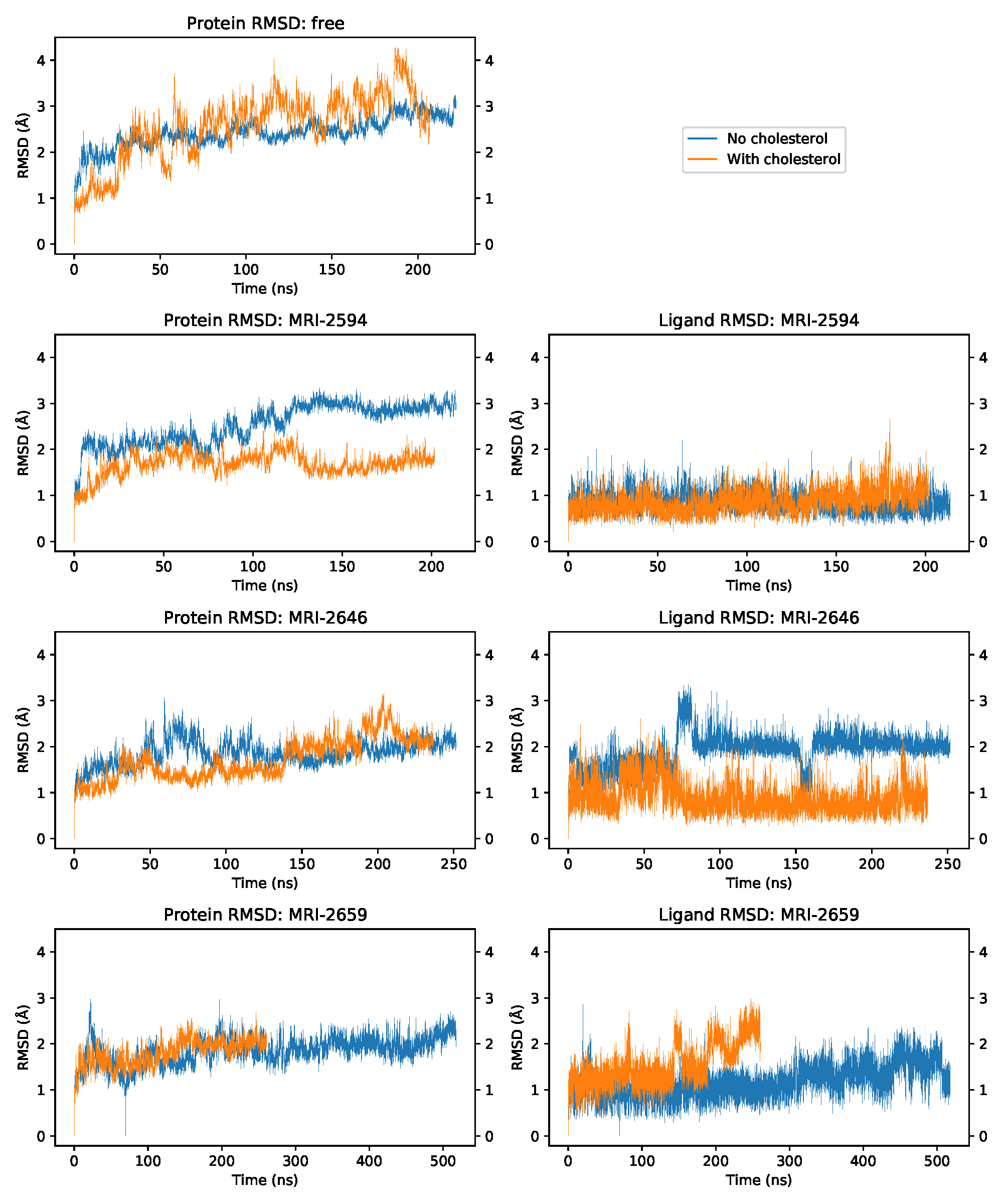


**Supplementary Figure 7**. RMSD of CB_2_ protein C-alpha atoms (top) and ligand (bottom) over simulation time. Each simulation Note initial relaxation phase in each panel.


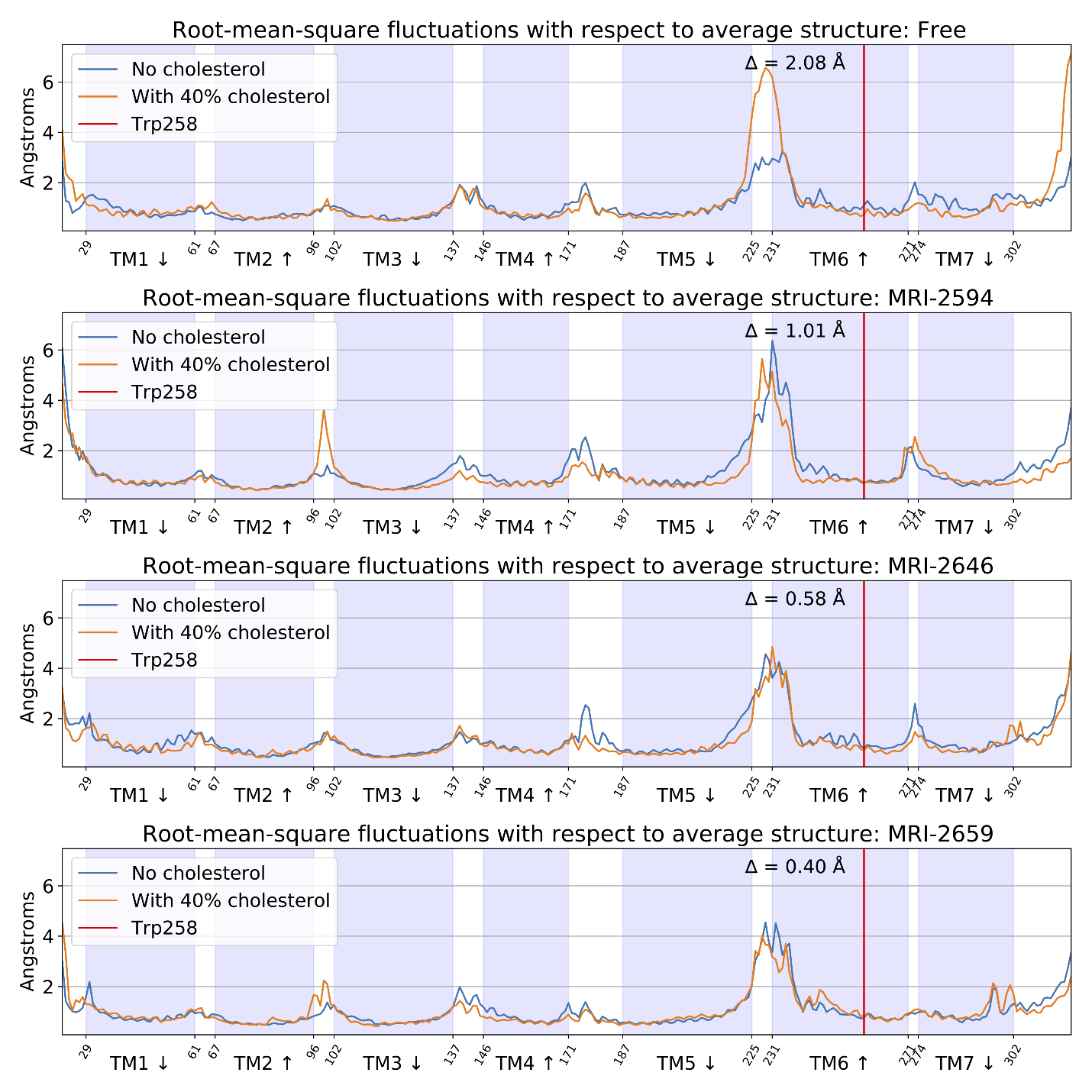


**Supplementary Figure 8.** Root-mean-square fluctuation of alpha carbons during equilibrium MD simulation, with respect to average structure, with and without 40 mol% cholesterol in membranes. The mean of absolute difference ("∆"), in the ICL3-TM6 region (residues 222–236) are listed in the respective graphs.
